# A protocol for a proof-of-concept randomized control trial testing increased protein quantity and quality in ready-to-use therapeutic food in improving linear growth among 6-23-month-old children with severe wasting in Malawi

**DOI:** 10.1371/journal.pone.0287680

**Published:** 2023-08-24

**Authors:** Isabel Potani, Allison I. Daniel, André Briend, Glenda Courtney-Martin, James A. Berkley, Wieger Voskuijl, Laura Vresk, Celine Bourdon, Sylvester Kathumba, Emmie Mbale, Robert H. J. Bandsma

**Affiliations:** 1 Translational Medicine Program, Research Institute, Hospital for Sick Children, Toronto, Canada; 2 Centre for Global Child Health, Hospital for Sick Children, Toronto, Canada; 3 Department of Nutritional Sciences, Temerty Faculty of Medicine, University of Toronto, Toronto, Canada; 4 Independent Nutrition Consultant, Toronto, Canada; 5 Centre for Child Health Research, University of Tampere School of Medicine, Tampere, Finland; 6 Department of Nutrition, Exercise, and Sports, Faculty of Science, University of Copenhagen, Copenhagen, Denmark; 7 Centre for Tropical Medicine and Global Health, University of Oxford, Oxford, United Kingdom; 8 Clinical Research Department, Kenya Medical Research Institute–Wellcome Trust Research Programme, Kilifi, Kenya; 9 Department of Paediatrics and Child Health, Kamuzu University of Health Sciences, Blantyre, Malawi; 10 Amsterdam Universtair Medische Centra, University of Amsterdam, Amsterdam Centre for Global Child Health, Emma Children’s Hospital, Amsterdam, The Netherlands; 11 Department of Nutrition and Human Immunodeficiency Virus, Ministry of Health, Lilongwe, Malawi; University of Agriculture Faisalabad, PAKISTAN

## Abstract

**Introduction:**

Ready-to-use therapeutic foods (RUTFs) have successfully promoted recovery from severe wasting and increased treatment coverage. However, RUTFs do not sufficiently improve linear growth, leaving many survivors of severe wasting at risk of persistent stunting, which is associated with high mortality risk, poor child development and non-communicable diseases in adulthood. High protein quantity and quality can stimulate linear growth.

**Aim:**

The trial aims to assess whether higher-protein-RUTF leads to higher concentrations of markers of linear growth compared to standard RUTF among 6–23 months old children with severe wasting.

**Methods:**

We designed a higher protein quantity and quality RUTF for a proof-of-concept (PoC) double-blind randomized controlled trial.

**Outcomes:**

The primary outcome is a change in insulin-like growth factor-1 (IGF-1), a hormone positively associated with linear growth after four weeks of treatment. Secondary outcomes include changes in ponderal and linear growth and in body composition from baseline to eight weeks later; plasma amino acid profile at four weeks; acceptability and safety.

**Implications:**

These findings will help in informing the potential impact of increased protein in RUTF on linear growth when treating severe wasting towards conducting a larger clinical trial.

**Trial registration:**

The trial has been registered on clinicaltrial.gov (NCT05737472).

## Introduction

In 2020, an estimated 13.6 million children aged 6–59 months suffered from severe wasting [1]. Severe wasting is defined as weight-for-length z-score (WLZ) <–3 standard deviations and/or mid-upper-arm circumference (MUAC) <115 mm [2]. Most children with severe wasting can safely be treated in their communities using ready-to-use therapeutic foods (RUTFs) and oral antibiotics without requiring admission to a hospital [3]. At enrolment into nutritional support programs, most children are both severely wasted and stunted (reportedly 69%), and while wasting improves with treatment, stunting does not [4]. Furthermore, some studies suggest that up to 38% of children who are not initially stunted are at risk of becoming stunted within a year following recovery from severe wasting [4, 5]. Stunting is linear growth faltering that is defined by a length-for-age z score (LAZ) <-2 SD [6]. Stunting is associated with long-term health outcomes, including poor child neurodevelopment, lower school achievement and income from work, and an increased risk of non-communicable diseases [7–10]. Improving protein quantity and quality intake during severe wasting treatment could improve linear and ponderal growth [11–14]. The current RUTF formulation recommended by WHO contains 10–12% energy from protein but falls short of protein quality requirements set by the Food Agricultural Organisation; with a Protein Digestibility Corrected Amino Acid Score (PDCAAS) of 0.83 that is below the ≥ 0.90 threshold [15, 16]. PDCAAS assesses protein quality in a food by the amount of essential amino acids against a standard amino acid score while correcting for the faecal amino acid digestibility. PDCAAS was superseded by the Digestible Indispensable Amino Acid Score (DIAAS), which is considered more accurate as it measures protein or amino acid digestibility at the terminal ileum and thought to better reflect amino acid absorption [15]. Designing a treatment product with optimal protein quantity and quality to aid in management of severe wasting and linear growth faltering is critical because children in recovery have higher protein requirements to support their need for linear and ponderal catch-up growth [17].

A sufficient and high-quality protein intake directly activates and upregulates growth factors, the Mechanistic Target of Rapamycin Complex 1 (MTORC1), growth hormone, insulin-like growth factor 1 (IGF-1), insulin-like growth binding protein 3 (IGFBP3), and peptide of type I procollagen (PICP) [12, 17–19] (S1 Fig). IGF-1 is a growth hormone released by the liver and muscles and circulates bounded to IGFBP3. Due to its role in growth, IGF-1 is gaining recognition as a biomarker of growth [20]. IGF-1 can also be stimulated by lactose, a carbohydrate in dairy, and by other bioactive compounds in dairy such as growth factors, cytokines, and hormones, thus, dairy products may also support growth and development through antimicrobial, anti-inflammatory, and immunomodulatory properties [20]. Proteins derived from dairy also have a high quality and digestibility score [21, 22]. Although there is biological plausibility that increased dietary intake of high-quality protein during the treatment of wasting could improve linear growth [23] the one previous study that tested this hypothesis found no impact [24]. The study was conducted among children with moderate wasting who were randomized to ready-to-use supplementary food (RUSF) that contained either protein sources from soy-isolate (low protein quality) or whey, a milk extract of high-quality protein. The lack of effect on linear growth could be attributable to both the lower amount of energy and protein in the whey RUSF (516 Kcal, 11.4g protein) compared to the soy-RUSF (559 Kcal, 17.1 g protein). Despite this initial finding, improved protein profiles in therapeutic foods used to treat wasting have been shown to positively affect body composition, weight gain, and recovery [24–26]. Therefore, this PoC l trial aims to assess the of higher protein quantity and quality in RUTF on linear growth and linear growth markers among young severely wasted children.

### Study objectives and hypothesis

The study’s primary objective is to assess whether higher protein quantity and quality increases circulating IGF-1 compared to standard RUTF after four weeks of treatment in 6–23-month-old children with severe wasting.

## Materials and methods

This protocol has been reported following the Standard Protocol Items: Recommendations for International Trials (SPIRIT) 2013 S1 Checklist [27].

### Trial design overview

A double-blind randomised controlled PoC trial designed to compare a novel RUTF formulation with increased protein quantity and quality (increased milk content and additional whey) to the current WHO recommended RUTF.

### Study setting

The PoC trial will be conducted in the Blantyre District of Malawi, where there are 27 outpatient clinics conducted at community health centres providing nutrition care under the supervision of the Blantyre District Health Office (DHO). The study will recruit from the four outpatient clinics with the largest catchment areas and, thus, with the highest number of admissions for uncomplicated severe wasting and/oedema. Between December 2018 and -December 2021, the admission at these clinics were: Bangwe (N = 325), Mbayani (N = 587), Ndirande(N = 315), and Limbe(N = 545). According to the Blantyre DHO, these health centres are in densely populated areas and are similar to the 8 other OTPs located in densely populated areas of Blantyre in terms of their organisation structure, staffing and severe wasting and/oedema admission phenotypes.

### Study population

Children aged 6 to 23 months enrolled to outpatient clinics with uncomplicated severe wasting (without oedema) and without medical complications, acute medical conditions, or lack of appetite.

### Sample size and sampling

The sample size for this PoC randomized control trial was calculated based on a study from Hoppe et al. (2004) [28]. The expected group difference in mean change of IGF-1 is 39.7 ng/ml with a standard deviation of 66 ng/ml [28] which yielded 46 children per arm for a power of 80% and alpha of 0.05. The sample size was then adjusted to account for 20% attrition and 10% mortality [29], resulting in 64 participants per study arm, and a total of 128 severely wasted children be recruited.

Participants will be identified using the convenience sampling method [30], where all children that meet the eligibility criteria at the selected outpatient clinics will be asked to participate. Additionally, in-depth interviews (IDIs) will be conducted with a subset of participants to assess the acceptability and use of the two RUTFs. The first recruited caregivers will be sequentially asked to participate in IDIs until 17 caregivers have completed the IDIs in each study arm to reach saturation of themes [31]. If theme saturation is not achieved after analysing the 34 IDIs, additional interviews will be conducted until saturation is reached.

### Eligibility criteria

Children will be eligible if aged 6–23 months with severe wasting defined as WLZ below -3 or MUAC below 115 mm without medical complications [3], newly enrolled to outpatient clinics on the recruitment day, able to feed orally in a normal state of health, the primary caregiver plans to stay in the study area for the duration of the study and their parent or guardian provides written informed consent.

Children will be excluded if they have edematous malnutrition, chronic conditions including cerebral palsy, tuberculosis, are HIV infected or exposed, have a terminal illness such as cancer, had severe malnutrition in the prior eight weeks, were previously enrolled to this trial, are participating in other nutrition or health intervention studies or have a known intolerance or allergy to any ingredients of the investigational products. The eligibility criteria were determined to exclude any medically complicated children e.g., children with oedema considering that this is a PoC trial that strived to have a small sample size [32–34]. Including children with nutrition oedema may dilute the effect of the intervention, and thus require a large sample size [35].

### Interventional products

The products have already been produced and are isocaloric, but the higher protein RUTF has a higher proportion of milk powder and whey protein. The new formulation has higher protein quantity and quality (Table 1) providing 15% of total energy from protein compared to 10% in standard RUTF. The DIAAS of the high-protein RUTF is 1.18 compared to 0.76 of the standard RUTF whereas the PDCAAS values are 1 and 0.83 respectively. The PDCAAS and DIAAS were calculated using faecal digestibility of protein and ileal digestibility of essential amino acids respectively in the ingredients based on studies conducted in pigs [15, 21, 36]. The PDCAAS was truncated to 1 whereas DIAAS was not truncated to 1 as per the respective calculation guidelines [15, 16].

**Table 1:** Nutritional composition of standard [control] and higher protein RUTF.

		HIGHER PROTEIN RUTF	STANDARD RUTF
Components	Unit	Per sachet(92g)	Per sachet (92g)
Calories	Kcal	506	
**Protein energy**	**% of total energy**	**15**	**10**
Proteins	% by weight	19	13
**DIAAS/PDCAAS**		**1.18/1**	**0.76/0.85**
**Essential amino acids**			
Histidine	g	0.6	0.3
Isoleucine	g	1.0	0.6
Leucine	g	1.7	1.0
Lysine	g	1.5	0.7
Methionine	g	0.4	0.2
Phenylalanine	g	0.8	0.6
Threonine	g	0.9	0.5
Tryptophan	g	0.3	0.2
Valine	g	1.1	0.6
**Non-essential amino acids**			
Alanine	g	0.7	0.5
Arginine	g	0.9	
Aspartic acid	g	1.7	1.2
Cystine	g	0.4	0.1
Glutamic acid	g	3.6	2.5
Glycine	g	0.5	0.4
Proline	g	1.1	0.8
Serine	g	0.9	0.6
Tyrosine	g	0.8	0.5
Met/Cys	-	1.0	1.5
Phe/Tyr	-	1.0	1.5
Carbohydrates	% by weight	32	39
Sucrose	% by weight	16	
Sucrose energy	% of total energy	12	
Lipids	% by weight	33.3	33.0
Lipids energy	% of total energy	59	
Linoleic acid C18:2	% by weight	3.6	3.4
C18:2 energy	% of total energy	6.4	6.1
Linoleic acid C18:3	% by weight	0.7	
C18:3 energy	% of total energy	1.3	
Vitamins and minerals			
Calcium	Mg	416	
Copper	Mg	1.4	
Iron	Mg	11	
Iodine	μg	81	
Magnesium	Mg	101	
Manganese	Mg	0.2	0.3
Free phosphorus	Mg	348	395
Potassium	Mg	1196	
Sodium	Mg	140	
Selenium	μg	28	
Zinc	Mg	12	
Vitamin A	μg	829	
Vitamin B1	Mg	0.5	
Vitamin B12	μg	1.7	
Vitamin B2	Mg	1.7	
Niacin	Mg	5.2	
Pantothenic acid	Mg	3.1	
Vitamin B6	Mg	0.6	
Biotin	μg	62	
Folic acid	μg	209	
Vitamin C	Mg	52	
Vitamin D	μg	17	
Vitamin E	Mg α TE	21	
Vitamin K	μg	c17	

RUTF: Ready-to-use therapeutic food, DIAAS: Digestible indispensable Amino Acid Score, PDCAAS: Protein Digestibility Corrected Amino Acid Score

The daily RUTF dose is given according to body weight per the WHO 2013 guideline, to provide 150–220 Kcal/kg/day [3, 37]. The RUTFs will be provided for eight consecutive weeks.

In addition to the RUTFs, all children will receive routine care during outpatient treatment of severe wasting as per the Malawi Community Management of Acute Malnutrition (CMAM) guidelines [38]. This includes seven days of Amoxicillin for all children (50–100 mg/kg/day) prescribed at enrolment and Albendazole for children ≥ 12 months (200 mg for 12–23-month-olds; 400 mg for ≥ 24-month-olds) at the second week of enrolment. Children will also receive doses for missed vaccinations or a single dose of missed vitamin A supplementation (100,000 IU to 6–11-month-olds: 200,000 IU to 12–59-month-olds) as part of routine care.

### Randomization and treatment allocation

Each participant will be randomly assigned to either higher or standard protein RUTF. Sequential participant ID numbers will be generated and randomised using an established script in R (Version 4.2 or above) [39, 40] Block randomization by study site will be used to ensure equal random treatment allocation at each site. The block sizes will randomly vary between 1 to 6 participants. This will be conducted prior to study start by the co-Principal Investigator, who is not directly involved in recruitment. Each participant’s treatment assignment will be placed in opaque sealed envelopes to ensure allocation concealment.

### Blinding of treatment

This study will be double blinded as the investigators (including all study staff) and participants will be blinded from the treatment allocation until data analysis is complete. This strategy minimizes the risk of bias due to awareness among participants and investigators of the group receiving the higher protein quantity and quality RUTF [41].

The two RUTFs are similarly packaged in 92g sachets, differing only by colour and code number (i.e., grey-88 or purple-99). As much as possible, the RUTFs have been matched for colour, texture, and smell; and based on tasting tests at the factory, no significant differences were noted. Children not recruited in the study will receive standard RUTF supplied from the health facility which is packaged in red and white.

### Informed consent

Children seeking treatment at any participating outpatient clinics will be screened for eligibility by study fieldworkers (S1 Table). Once their eligibility is confirmed, a fieldworker will proceed to ask caregivers for consent by confirming that the caregiver is the child’s primary guardian and verbally explaining the details of the study in the local language, Chichewa. The fieldworker will then verbalise to the caregiver all written information included in the informed consent form as adult illiteracy is high in Malawi. Caregivers will be provided time to read or ask questions regarding the study and/or the consent form before being asked to sign. Where a caregiver cannot read or write, an impartial literate witness identified by the caregiver will be asked to document that consent has been provided. A written signature or thumbprint of the parent/guardian will mark consent to participate in the study. If the caretaker cannot or chooses not to participate in the study, the child will receive standard nutrition care at the outpatient clinic. The research team have been certified in Good Clinical Practice (GCP) and further trained in the standard operating procedure for obtaining consent using didactic learning and role plays.

### Withdrawal from the study

Participants will be removed from the study if the caregiver decides to withdraw. A caregiver’s choice to withdraw will not prejudice the child against receiving routine treatment. Participants may also be withdrawn if there are clinically relevant signs, symptoms, or adverse events that, in the opinion of the Principal Investigator, warrant withdrawal for the safety of the participant. Participants may also be withdrawn (without unblinding) if the participant is enrolled in violation of the study protocol or on the advice of the data safety and monitoring board (DSMB).

Participants that withdraw from the study will be referred to routine health services as indicated by the child’s medical and nutritional condition. They will also be invited to seek medical attention at the study clinic for any new symptom until 60 days after enrolment. Participants who withdraw are not replaced. Data from withdrawn participants will remain in the study database and used, if possible, for final analyses unless parents/guardians request removal. Furthermore, samples collected before withdrawal will be used as planned unless parents/guardians request that samples be destroyed without analyses. The reasons for withdrawal will be recorded.

### Data collection

**Fig 1:**
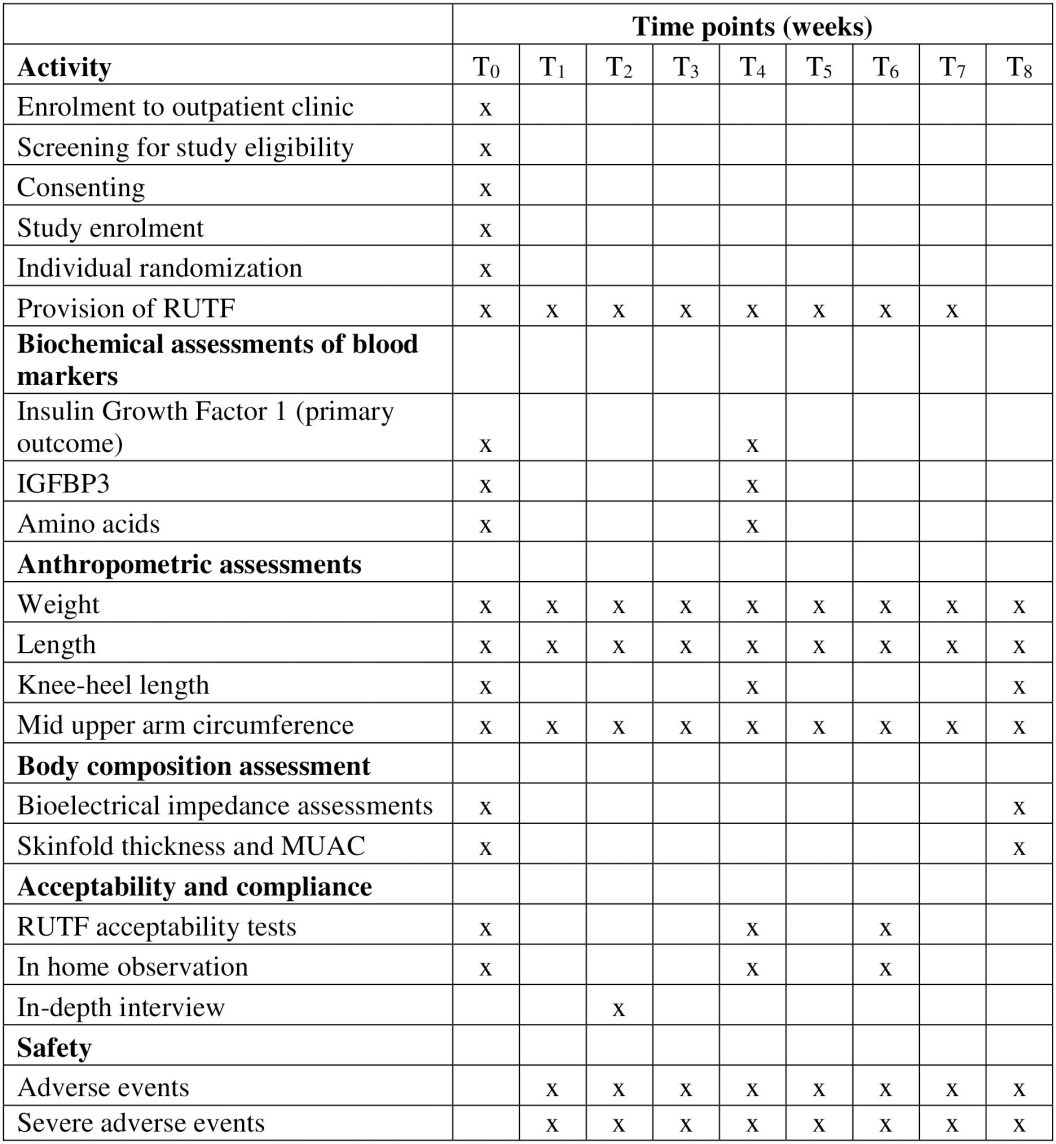
Data collection time points.

The research clinician and fieldworkers will collect data at the time points specified in Fig 1. Caregivers will be asked to return with their infants to the outpatient clinic to obtain weekly rations of RUTF for eight consecutive weeks. The eight-week duration was selected as most children with SAM are successfully treated within this timeframe [42]. The caregivers are reminded of the follow-up visit by phone (call or text message), and those who miss an appointment will be traced at home through visiting fieldworkers. If the prespecified time point is missed, attempts will be made to collect the data within five days. Participants will be marked as lost to follow-up if they consistently miss protocol study visits, are not reachable by telephone or any other means of communication and/or cannot be located until a week after the final visit at week 8.

### Assessment of outcomes

The trial outcomes are defined in Table 2; these include biomarker, anthropometric, body composition, biochemical, acceptability, compliance, and safety outcomes. The four-week timepoint to assess changes in IGF-1 and IGFBP3 was selected based on evidence that within this period both RUTF intake and rate of ponderal growth are at their highest [43]. Also, early increments in IGF-1 and IGFBP3 at 4-weeks have been associated with increased linear growth at 12 weeks [44, 45].

**Table 2:** Outcomes of interest.

Primary Outcome	Description	Statistical analysis
Change in IGF-1	1. Change in IGF-1 (ng/ml) from baseline to the 4^th^ week of follow-up (continuous).	Unadjusted and adjusted generalised linear mixed- effects models
**Secondary outcomes**		
Anthropometry	**Linear growth to 8 weeks** Change in length-for-age z-score (continuous). Change in length (cm/day) (continuous) Change in knee-heel length (mm/day) (continuous) **Ponderal growth to 8 weeks** Weight gain velocity in (grams per kg body weight per day) Change in Weight-for-age z-score (a composite measure of linear and ponderal growth) Change in Weight-for-length z-score (a measure of wasting) Change in mid-upper arm circumference mm (a measure of wasting) MUAC gain mid-upper arm circumference mm/week	Unadjusted and adjusted^1^ generalised linear mixed- effects models
Body composition	Change in fat-free mass (FFM) and fat mass (FM) using skinfold thickness and BIA to 8 weeks.	Unadjusted and adjusted generalised linear mixed- effects models
Other treatment outcomes	Wasting Recovery: proportion of children cured, defined as mid-upper arm circumference ≥12.5 mm or WLZ≥ 2, clinically well at 8 weeks following enrolment. Severe wasting Recovery: proportion of children cured, defined as mid-upper arm circumference≥11.5 mm or WLZ≥ 3, clinically well at 8 weeks following enrolment. Defaulting: proportion of children who are absent for two consecutive visits Non-responders: proportion of children with a MUAC <12.5 mm or WLZ <2 by 8 weeks of treatment Relapse: proportion of children who were cured: ≥12.5 mm or WLZ ≥2; and developed severe wasting again: < 12.5 mm or WLZ <2 in the 8-week follow-up period Mortality: proportion of children who died by 8 weeks post-enrolment	Unadjusted and adjusted^1^ generalised logistic mixed-effects models and weeks to recovery will be compared using mixed-effects survival analysis.
Growth modulation mechanism	Change in plasma essential amino acids after 4 weeks of RUTF intake from baseline (each amino acid and derived ratios and summary variables). Change in Plasma IGFBP3 after 4 weeks of RUTF intake from baseline (ng/ml)	Unadjusted and adjusted^1^ Mixed effects generalised linear models.
Compliance	1. Difference between RUTF ration provided and empty RUTF sachets returned at follow up. 2. Number of missed visits Number of late visits beyond + 3 days.	Independent t-test and Mann-Whitney U Test when the outcome is assessed as a ordinal categorical variable or a Poisson model for count data. These data will be used to construct an exploratory composite latent score to represent participant compliance.
	3. In-depth interview to assess if RUTF is given according to prescribed dose	Thematic analysis technique
	4. In-home observation to assess if RUTF is given according to prescribed dose	Chi-square test and Fischer’s Exact test when the outcome is assessed as a categorical variable
Acceptability	1. Intake of prescribed RUTF adapted from the Action Contre la faim appetite test [45]	Chi-square test and Fischer’s Exact test when the outcome is assessed as a categorical variable
	2. In-depth interview on mothers’ perception of role and use of RUTF	Thematic analysis technique
	3. In-home observation to assess amount of RUTF eaten by the child	Thematic analysis technique and Mann-Whitney U Test
Adverse Events	Weekly assessments of current or previous (last 7 days): Fever Anaphylaxis Allergy Rash Diarrhoea Cough Health care visits Any illness reported by caregiver or observed by the research nurse	Negative binomial regression on individual and overall event counts. and time-to-adverse event (survival) analysis on each adverse.
Severe Adverse Events	Any occurrences that result in death or are life-threatening. Events that result in persistent/ significant disability or hospitalization for any reason are also considered SAEs. Any medical event leading to severe clinical deterioration that requires medical or surgical intervention to prevent one of the SAEs listed above is also considered an SAE	2x2 contingency tables with fisher exact test.

#### Anthropometry

The study personnel will be trained in anthropometric assessments to identify and treat severe wasting as recommended by WHO [2, 46]. Weight, length and MUAC will be measured at every outpatient clinic visit. For weight, a single measurement will be taken using a paediatric weighing scale (ADE M112600U) to the nearest 100 g. Length will be measured using an infant length board (SECA 416) to the nearest 0.1 cm; two measurements will be taken. If the difference is greater than 0.5 cm, a third measure will be taken. The average of the two closest length measurements will be considered the final length. MUAC will be measured using a non-elastic MUAC band (UNICEF-S0145620) to the nearest 0.1 cm; two measurements will be taken with a maximum limit of 0.5 cm; if the difference is greater than 0.5 cm, a third measure will be taken. WLZ will be calculated using the WHO field growth charts on the day of enrolment [2]. The WHOAnthro program will be used to calculate z-scores for data analysis [47]. The participants’ knee-heel length [48] will be measured at enrolment and at eight weeks, as leg length is more responsive to environmental stimulants than full body length [49]. Knee-heel length will be measured using an Absolute Digital Digimatic Vernier Caliper with a resolution of 0.01 mm (Mitutoyo: 500-197-20/30 200mm/8) mounted with knee and heel caps on the left leg. During the measurement, the child will be seated with both legs hanging over the edge of a table or the caregiver’s lap. The measurer will place the fixed arm of the Caliper under the heel using the left hand. Using the right arm, the measurer will move the calliper’s measuring handle until it touches the lateral condyle of the knee and apply firm pressure. The reading will then be taken by pressing the black trigger button on the Caliper with the right thumb. The distance between the knee (from the lateral condyle) and the heel (calcaneus) will be measured five consecutive times. The average of the five measures will be considered the final knee-heel length.

#### Biochemical assessments

A research nurse will collect 3ml venous blood samples at enrolment and week four. These are primarily for assessing IGF-1, IGFBP3 and essential amino acid profiles which are positively associated with growth [12]. The plasma will be aliquoted and stored (-80*°C*) at a Biochemistry laboratory at Kamuzu University of Health Sciences (KUHeS), College of Medicine campus, pending analysis.

*Laboratory analysis*. IGF-1 and IGFBP3 will be analysed using the commercial MILLIPLEX^®^ MAP HIGF-I, II Magnetic Bead Panel Kit (catalogue no. HIGFMAG-52K; EMD Millipore) and MILLIPLEX^®^ MAP HIGFBP, Magnetic Bead Panel Kit (catalogue no. HIGFMAG-53K; EMD Millipore), respectively. According to the manufacturer’s instructions, plasma will be diluted and incubated with the antibody-coupled microspheres and then with biotinylated detection antibody before adding streptavidin–phycoerythrin. The captured bead complexes will be measured with the Bio-Plex^®^ 200 system (Bio-Rad Laboratories). The detection ranges of the IGF-1 and IGFBP3 assays are 0.12–88.2 ng/mL and 0.07–50ng/mL respectively. These bead panels were selected as they are more sensitive and specific compared to commonly used standard enzyme-linked immunosorbent assay. Amino acid profiles will be assessed using mass spectrometry following mass spectrometer manufacturers instruction. The mass spectrometer of choice is yet to be identified.

Exploratory analyses that may be conducted include other growth biomarkers such as measures of inflammation e.g., C-reactive protein (CRP), cartilage growth markers e.g., PICP, and other IGFBPs [45, 50].

#### Body composition assessment

The PoC trial will assess body composition using triceps skinfold thickness (TSF), MUAC and single-frequency bioelectrical impedance assessment (BIA). These are accepted body composition tools that are feasible to conduct in field settings due to their low cost and relative ease of use [51, 52].

TSF will be measured, using a skinfold calliper (Harpenden 22-SFC-0112), and the equations below will be used to estimate body composition using TS [51, 52].

1. Total arm area (TUA) = *MUAC*^2^/4π
2. Upper arm fat area = *MUAC*/(*TS*/2)
3. Upper arm muscle area estimate = *TUA* − *UFE*
4.
$\%\;\text{of}\;\text{fat}\;\text{in}\;\text{the}\;\text{upper}\;\text{arm}\;\text{area}=(\frac{UFE}{TUA})\times 100$

BIA will be measured using a Bioelectrical Impedance Analyzer (BodystatQuadScan4000) at 50 ‘Hz as per the manufacturer’s instructions and as we and others have previously performed among children with severe wasting [52]. The child will be placed in a supine position, with limbs spread, without wet or soiled diapers, and preferably at rest. Three repeat measurements will be collected for resistance (*ohms*), reactance (*ohms)*, and phase angle (*degrees*). A maximum difference of 10 ohms among measurements for resistance and reactance will be allowed, else, the assessments will be repeated. Fat-free mass (*kg*), fat mass (*kg*), total body water (*L*), extra-cellular water (*L*), Intra-cellular Water (*L*) and Body Cell Mass (*kg*) will be obtained from automated outputs, but these will be verified during data analysis as a quality assurance measure as described before [52].

Training and rigorous quality control measures (i.e., conducting repeat measurements with maximum limit of agreement) will promote validity of the anthropometric and body composition measures, monthly refresher training will be conducted through team review of standard operating procedures (SOPs). Furthermore, data quality checks will be performed by the Co-PI on a weekly basis to identify implausible trends such as declines in length and extreme weight gains between timepoints. Additionally, weighing scales will be calibrated daily by weighing a 1kg weight prior to use. The Bioelectrical Impedance Analyzer (BodystatQuadScan4000) will be calibrated as per manufacturer instruction, which includes maintaining software updates, ensuring electrodes and cables are clean and intact and conducting test measurements with metal of known conductivity. Body composition assessment will be ideally conducted by one fieldworker to minimise intra-observer variability.

### RUTF acceptability and compliance

To assess the acceptability of the novel RUTF, appetite tests will be conducted at every weekly visit based on a protocol designed by Action Contre la Faim and WHO [53], as shown in Table 3. Children who fail the test will be referred to the health facility’s clinicians to be evaluated for inpatient care eligibility.

**Table 3:** Appetite test.

Steps in conducting an appetite test	
1. The appetite test should be conducted in a separate quiet area. 2. Explain to the caregiver the purpose of the appetite test. 3. Explain how it will be carried out. 4. The caregiver should wash her hands. 5. The caregiver should sit comfortably with the child on his lap. 6. The caregiver should offer the RUTF from the packet or put a small amount on her finger and give it to the child. 7. The caregiver should offer the child the RUTF gently, encouraging all the time. If the child refuses, then the caregiver should continue to quietly encourage the child. She can take time for the test. The child must not be forced to take the RUTF 8. The child needs to be given plenty of water from a cup as he/she is taking the RUTF	
Assessment of appetite test outcome	
Child weight	Test passed if the child eats this amount, or more
Less than 4 kg	1/8 of the sachet
More than or equal to 4 kg, but less than 7 kg	1/4 of the sachet
More than or equal to 7 kg, but less than 10 kg	1/3 of the sachet
More than or equal to 10 kg, but less than 15 kg	1/2 of the sachet
More than or equal to15 kg, but less than 30 kg	3/4 of the sachet
More than or equal to 30 kg	1/1 of the sachet

To assess acceptability at home, observed intake of the RUTFs will be conducted two weeks post- recruitment by a study fieldworker. The two-week post-recruitment time point was selected as rapid weight gain is expected within the first 2–4 weeks of treatment [43]. The study fieldworker will observe the amount of RUTF provided to the child between 8 am and 12 pm and assess whether the intake corresponds with the prescribed RUTF. The fieldworker will also observe how leftover RUTF is managed. After observing intake within the observation window, a questionnaire will be administered by the fieldworker to evaluate the caretaker’s perception, and thoughts regarding the child’s reaction based on a 5-point hedonic scale, where 1 = dislike very much, 2 = dislike, 3 = neither like nor a dislike, 4 = like and 5 = like very much. The scale is illustrated using a series of human face symbols with varying degrees of”smile” or”discontent“, a method previously used to measure acceptability of treatment foods in a similar population [54].

On the same day of home observations of RUTF intake, a study fieldworker will conduct and record an in-depth interview with the caregiver. Using a semi-structured guide, the fieldworker will assess the caregiver’s knowledge regarding the role and use of RUTF in the household. Specific areas of interest are the quantity and frequency of providing RUTF to the child, sharing practices within and outside households, selling of provided or leftover RUTF and any losses of RUTF at home.

At each clinic visit, caregivers will be asked to return empty and full sachets of RUTF to measure compliance.

#### Safety

We consider this nutrition intervention-based trial low risk given that these children have uncomplicated severe wasting. The intervention, higher protein RUTF, is considered safe as its formulation is based on standard WHO-approved RUTF formulation. The higher protein RUTF only differs in its’ protein quantity and quality and was designed to achieve protein intake amounts of children in high income countries.

Despite minimal safety concerns, data on adverse events (AEs) will be collected throughout the follow-up period and reported. Severe AEs include allergy or anaphylaxis, hospitalisation and mortality during study follow up. At every visit, the research nurse will perform a routine physical examination on the children to assess current morbidity and will ask the caregiver if the child experienced illness in the previous week that may have resolved. Attempts to contact children lost to follow-up will be made to assess the vital status. The cause of death for any deaths occurring within the follow up duration will be assessed and recorded using a verbal autopsy form adapted from the Child Acute Illness Nutrition Network [55].

Children requiring inpatient care will be referred to inpatient treatment facilities as per local treatment guidelines. This and other life-threatening events, severe clinical deterioration, any persistent/significant disability, and urgent treatment given to prevent any of these outcomes will be reported as an SAE. Children who experience SAEs will be followed up by the research nurse until their resolution or stabilization. The PIs and one co-investigator will assess the relationship of all SAEs with the higher protein RUTF within 24 hours of identifying an occurrence. This procedure will follow the World Health Organisation-Uppsala Monitoring Centre causality assessment scale which assesses the type of event, the relationship between the event and timing of RUTF administration, and the known biology of the intervention product [56, 57].

Emergency unblinding may be undertaken if beneficial to the clinical treatment of the participant. Unblinding will not be undertaken to determine if a child should continue receiving the investigational product or if the investigational product has been stopped and knowledge of the allocation would not affect other treatments.

### Incidental findings

The investigators will inform the respective participants of any incidental findings identified during the study and provide referrals for further medical care. The study sponsor and ethical regulatory bodies will also be notified of these findings.

### Trial status

The trial was submitted at clinicaltrials.gov for registration on 10 November 2022, and was accepted on 2 January 2023 (NCT05737472). Participant recruitment begun before this process was finalised (14 November 2022) to mitigate loss of investigational product whose expiration date was 30 April 2023. At the time of protocol publication, the authors confirm that all ongoing and related trials for this drug/intervention are registered. The trial anticipates a recruitment period of 6 months. An additional nine-month period is anticipated for laboratory, data analysis, and dissemination.

### Data management and analysis

All participants will be assigned a study identification number at enrolment that will be used on all the data collection forms, helping ensure privacy. The anonymized data will be entered into a secure, password-protected Research Electronic Data Capture (REDCap) database accessed by members of the study team only [58]. Once recruitment and laboratory analysis are complete, data will be analysed using R (Version 4.2 or above) [39, 40]. Using an intention to treat analysis approach, differences between study arms will be assessed using generalised linear mixed effect models to analyse differences (mean change) in the primary and quantitative secondary outcomes measures between baseline and follow up measures at four and eight weeks(Table 2). These models were selected because they can fit different regression types (e.g., Gaussian, Logistic, Poisson) and have fewer assumptions regarding normality and independence of measurements (i.e., can account for repeated measures within participants) and can handle missing covariate data avoiding row-wise deletion. As this is a randomized control trial, we expect an equal distribution of confounders between arms; thus, we will report unadjusted analyses as the main analyses. However, chance imbalances for important baseline covariates are possible and can be seen even in large clinical trials [59, 60]. Thus, as a sensitivity analysis, we will run models with two-levels of adjustment: 1) accounting for participant’s sex, age, and site and 2) with further adjustment considering baseline weight-for-age, socioeconomic status, small birth size and breastfeeding status [61]. Level-II adjustment factors will only be retained if they significantly improve model fit and coefficients of the base unadjusted models. Level-I adjusted, and level-II adjusted models will be presented using forest plots and compared using fit metrics. These covariates were selected because studies have reported them to be associated with growth or growth markers and can confound the intervention effect [61–64]. All covariates will be collected at enrolment while age will be calculated for every visit as the difference between the date of birth (obtained from the child’s health card or maternal recall) and the date of the OTP visit. Additional sensitivity analyses include conducting a per protocol analysis, including only the children that complete the planned treatment, adjusting the analysis for counts of unreturned sachets and, as an exploratory approach, adjustment for study compliance using a latent composite score built using number of missed or late visits (+3 days), together with counts of unreturned sachets. Depending on distribution, the score may be used categorised.

A written analysis plan will be reviewed and approved by the DSMB prior to undertaking the analysis.

The data collected from the in-depth interview will be transcribed in English verbatim and then written for analysis. The thematic analysis technique with a deductive coding approach will be employed to analyse the data [65]. All the audio recordings will be kept secured and accessible to only the research team. After completion of the trial, the qualitative data will be analysed using NVivo 10.

### Trial governance

The trial has a DSMB comprising three experts in malnutrition treatment, statistics, and ethics. The DSMB met at the beginning of the trial to evaluate the protocol and study processes. The DSMB will review the study outcomes and SAEs at the mid and endpoint of the trial. The trial does not have a trial steering committee.

### Ethical considerations

Ethical approval was obtained from the National Health Sciences Research Committee (NHRSC) at the Malawi Ministry of Health (reference number:21/11/2827) and the Research Ethics Board at the Hospital for Sick Children in Canada (reference number:1000079230). Prior to implementation, any changes made to the study protocol will be reviewed and approved by the principal investigator and the relevant ethical bodies. In addition, the study trial registry and published protocol will be updated to reflect the changes.

The study procedures require participants to attend more outpatient clinic visits and spend for each visit, a longer time than they would if not enrolled in the study. For this reason, participants will be given an allowance of 5000 Malawi Kwacha (USD5) at the time of enrolment and of graduation. Additionally, the participants will be given 1000 Malawi Kwacha (USD1) for each study visit based on minimum compensation and reimbursement fees for research in Malawi.

Study participant identification numbers will be used on all data collection forms to maintain participant anonymity. All paper data collection tools with participant information will be kept under a locked cabinet at the main study office.

## Discussion

There is a need to improve the health outcomes for children recovering from severe wasting. Presently, the rate of weight gain, catchup linear growth and developmental outcomes are considered suboptimal for children recovering from severe wasting [4, 5, 7–9]. The study findings could provide insight into correcting stunted linear growth, which could improve poor developmental outcomes associated with poor linear growth [66]. Although there is evidence that poor protein intake is associated with stunting [67], this is the first study to assess the potential of a higher protein quantity and quality RUTF in improving growth and body composition versus the standard RUTF formulation [23].

The optimal daily protein intake to achieve ponderal and linear growth recovery during SAM treatment is unclear. For our study, the protein content in higher protein RUTF was driven by our aim to increase protein quality (i.e., to achieve a DIAAS or PDCAAS>0.9). To ensure safety of increased protein intake with the higher protein RUTF, we consulted food technologists and medical doctors experienced in treating severe wasting. The protein levels were deemed safe and unlikely to strain kidney function. Additionally, this PoC trial excludes severely ill children (e.g., children with infections, nutritional oedema, kidney, or liver diseases) who are more at risk of severe metabolic derangement that could be worsened by high protein intake [32]. The safety outcomes reported by this PoC trial will help advance the evidence regarding the safety of using higher protein diets during severe wasting treatment.

The study’s PoC design uses a proxy marker for linear growth, IGF-1, as a primary outcome [33] in a phase II clinical trial. To observe changes in linear growth, larger numbers and at least 12 weeks of follow-up would have been required given measurement errors and small detectable differences between groups [68, 69]. This PoC was designed to efficiently determine if larger follow-up phase III trials powered for longitudinal growth are warranted. This is a useful approach when assessing interventions that may be costly and whose benefits are unknown.

The PoC trial has several limitations which could be addressed by future studies, the trial does not assess child development outcomes, NCD risk and does not collect any markers for gut microbiome. These decisions were made based on cost-benefit, resource availability, and the effort to minimize the study burden on participants. In terms of study burden, the participants will be subject to responding to questionnaires, blood sampling, and repeat anthropometric measurements, body composition assessment, RUTF acceptability testing, AEs and SAEs evaluation and home visits for some participants.

While the exclusion of medically complicated children such as, any oedema, HIV infected and HIV exposed, and recent history of complicated SAM can limit the generalizability of the findings, we chose to minimize any risk of metabolic disturbances in these children that may arise from high protein intake. Furthermore, this PoC trial was not designed to compare cost-effectiveness of the products. Recognising that the high cost of RUTF has been attributed to dairy components, cost-effectiveness is an important outcome that should be considered in the full clinical trial [70, 71]. Also, the mortality rate used to adjust the sample size is potentially overestimated as it was based on findings from an early study on home-based severe acute malnutrition treatment in Malawi, which included children having a recent history or complicated SAM, existing or resolved nutrition oedema, and HIV infection [29].

Another important consideration is that interventions like higher protein RUTF aim to promote rapid weight gain and, while this has been shown to be favourable for outcomes such as mortality, muscle strength and height gain; there is emerging evidence suggesting that rapid weight gain is also associated with a higher NCD risk later in life [72]. Thus, the balance of risk to benefits of these interventions must contextualize both short and longer-term outcomes.

The trial findings will be reported following the Consolidated Standards of Reporting Trials (CONSORT) guidelines [73, 74] and published in an open-access peer-reviewed journal regardless of positive or negative results to avoid publication bias. The results will also be shared with the NHRSC, relevant health forums in Malawi and at international scientific meetings. De-identified data may be made available in line with the study’s sponsor’s procedures for release.

## Supporting information

S1 Checklist SPIRIT 2013 checklist: Recommended items to address in a clinical trial protocol and related documents*. (DOC)

S1 Fig Role of protein quality and quantity in growth stimulation. (TIF)

S1 Table Study team composition. (DOCX)

S1 File (DOCX)

S2 File (DOCX)
