# Supplementary figures and images for: A protocol for a proof-of-concept randomized control trial testing increased protein quantity and quality in ready-to-use therapeutic food in improving linear growth among 6-23-month-old children with severe wasting in Malawi
Source: PLoS One. 2023 Aug 24;18(8):e0287680. doi: 10.1371/journal.pone.0287680 (PMC10449476; doi:10.1371/journal.pone.0287680)

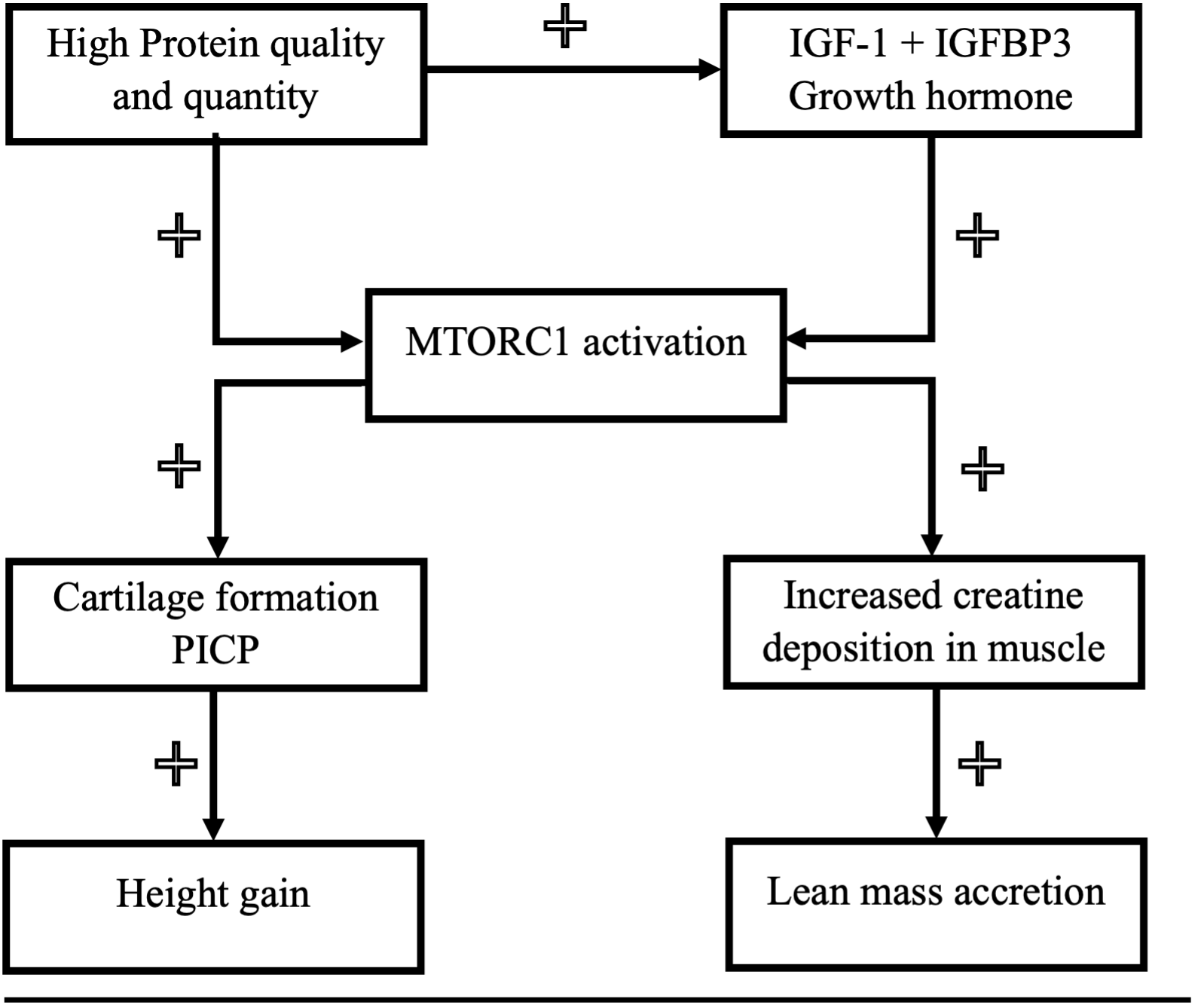

Supplement: S1 Fig — (TIF) [file pone.0287680.s002.tif]
