# Supplementary material for: A protocol for a proof-of-concept randomized control trial testing increased protein quantity and quality in ready-to-use therapeutic food in improving linear growth among 6-23-month-old children with severe wasting in Malawi
Source: PLoS One. 2023 Aug 24;18(8):e0287680. doi: 10.1371/journal.pone.0287680 (PMC10449476; doi:10.1371/journal.pone.0287680)
Supplement: S1 Table — (DOCX) [file pone.0287680.s003.docx]

**Supplementary Table 1: Study team composition**

| **Principal Investigator** | |  |
| --- | --- | --- |
| **Name and title** | **Main institutional affiliation** | **Location** |
| Dr. Robert Bandsma | Associate Professor of Paediatrics and Gastroenterologist  The Hospital for Sick Children,  Toronto,  Canada. | Canada |
| **Co-Principal Investigator** | | |
| **Name and title** | **Main institutional affiliation** | **Location** |
| Ms. Isabel Potani | PhD Candidate  Department of Nutritional Sciences,  University of Toronto  Canada. | Malawi and Canada |
| **Co-Investigators: Study design and conceptualisation** | | |
| **Name and title** | **Main institutional affiliation** | **Location** |
| Dr. Emmie Mbale | Paediatrician  Queen Elizabeth Central Hospital  Blantyre, Malawi | Malawi |
| Dr. Allison Daniel | Nutritionist  University of Toronto  Department of Nutritional Sciences,  Canada. | Canada |
| Ms. Celine Bourdon | Data analyst/Laboratory Research Manager,  PhD candidate  The Hospital for Sick Children,  Toronto  Canada | Canada |
| Dr Glenda Courtney-Martin | Assistant Professor  The Hospital for Sick Children  Canada | Canada |
| Ms Laura Vresk | Research Fellow & Clinical Dietitian  The Hospital for Sick Children,  Toronto  Canada | Canada |
| Dr. André Briend | Adjunct Professor  Tampere University,  Finland | France |
| Mr. Sylvester Kathumba | Nutritionist  Department of Nutrition and HIV  Malawi | Malawi |
| Dr. Wieger Voskuijl | Paediatrician  Amsterdam Centre for Global Child Health,  Emma Children’s Hospital,  Amsterdam University Medical Centre,  The Netherlands | Netherlands |
| Dr. James Berkley | Professor Of Paediatric Infectious Diseases  Nuffield Department of Medicine,  University of Oxford,  United Kingdom | Kenya |
| **Data collection Team** | | |
| **Name and title** | **Main institutional affiliation** | **Location** |
| Mr. Chisomo Eneya | Study Clinician/Coordinator  Friends of Sick Children  Kamuzu University of Health Sciences, Malawi | Malawi |
| Mrs Agnes Malamula | Study Nurse  Friends of Sick Children  Kamuzu University of Health Sciences, Malawi | Malawi |
| Ms Takondwa Mbulaje | Study Fieldworker  Friends of Sick Children  Kamuzu University of Health Sciences, Malawi | Malawi |
| Mr Abel Tembo | Study Fieldworker  Friends of Sick Children  Kamuzu University of Health Sciences, Malawi | Malawi |
| Mr Frank Tembo | Study Fieldworker  Friends of Sick Children  Kamuzu University of Health Sciences, Malawi | Malawi |
