# Supplementary material for: A protocol for a proof-of-concept randomized control trial testing increased protein quantity and quality in ready-to-use therapeutic food in improving linear growth among 6-23-month-old children with severe wasting in Malawi
Source: PLoS One. 2023 Aug 24;18(8):e0287680. doi: 10.1371/journal.pone.0287680 (PMC10449476; doi:10.1371/journal.pone.0287680)
Supplement: S1 File — (DOCX) [file pone.0287680.s004.docx]

**Protein + trial: Patient information and consent form**

**Study title: A proof-of-concept randomized control trial on the role of higher protein quantity and quality-ready-to-use therapeutic food in improving linear growth among 6–23-month-old children with severe wasting. “The Protein + trial”**

**INVESTIGATORS**

| **Principal Investigator** | |
| --- | --- |
| Dr. Robert Bandsma | Associate Professor of Pediatrics  The Hospital for Sick Children  Canada |
| **Co-Principal Investigator** |  |
| Ms. Isabel Potani | PhD Candidate  University of Toronto (Department of Nutritional Sciences), Canada, and College of Medicine Malawi  Canada |
| **Co-Investigators** | |
|  |  |
| Dr. Allison Daniel | Independent nutrition consultant  Canada |
| Dr. Glenda Courtney-Martin | Assistant Professor  The Hospital for Sick Children  Department of Nutritional Sciences  Canada |
| Laura Vresk | Research Fellow & Clinical Dietitian  The Hospital for Sick Children  Canada |
| Dr. André Briend | Adjunct Professor  Tampere University.  Finland |
| Mr. Chisomo Eneya | Study Clinician  Childhood Acute Illness Nutrition Network  Kamuzu University of Health Sciences, Malawi |
| Mr. Sylvester Kathumba | Nutritionist  Department of Nutrition and HIV  Malawi |
| Dr. Wieger Voskuijl | Paediatrician  Amsterdam Centre for Global Child Health, Emma Children’s Hospital, Amsterdam University Medical Centre  The Netherlands |
| Dr. James Berkley | Professor Of Paediatric Infectious Diseases  Nuffield Department of Medicine  University of Oxford |

You are being asked to allow your child to take part in a research study. The box below tells you important things you should think about before deciding/allowing your child to join the study. We will provide more detailed information below the box. Please ask questions about any of the information before you decide/allow your child to participate. You may also wish to talk to others (for example, your family, friends, or your doctor/nurse) about this study, before making a decision.

| **Key Information for You to Consider** |
| --- |
| - **Voluntary Consent**. Your child is being asked to volunteer for a research study. You can choose whether you/your child would like to participate or not. If you do agree you can change your mind at any time and withdraw your child from the research. This will not affect your child’s care now or in the future. - **Purpose**. We are conducting this research to understand whether giving higher protein quantity and quality RUTF can improve length or height gain in severely wasted children. Children with severe wasting have insufficient growth in length and height which may be due to insufficient protein in the standard RUTF formulation. - **Duration.** Your/your child’s participation in this study will last 2 months from the day of enrolment in the trial. - **Procedures and Activities.** Your child will first receive the medical attention if any. We will conduct assessments on your child for baseline information. This includes: - height, (lower leg length, weight, upper arm thickness (mid-upper arm circumference- MUAC) at every clinic visit for 8 weeks. Knee heel length will be measured at enrolment, week 4 and week 8 - Body composition assessment: skin fold thickness, body fat content using low electric current (bioelectrical impedance assessment) at enrollment, and week 8 - Blood tests: We will test for the following growth promoters: Plasma amino acids, insulin growth factor 1 (IGF-1) and insulin growth factor binding protein three (IGFBP3). We will take 5ml (about a teaspoon full amount) of blood on the week 0 (enrollment day) and on week 4 - Interviews and observation on RUTF intake and use at home and at the treatment facility - **Risks or disadvantages.** The formulation of the high protein RUTF that will be tested in this study is not very different from standard RUTF. The study’s RUTF formulation is only different in terms of protein quantity and quality, but the rest is the same. The high protein quantity and quality has been achieved by adding more milk. Therefore, the risks of harm are very low just as in the standard RUTF. Both the standard RUTF and high protein RUTF can cause allergic reaction in people with peanut allergies, although peanut allergies are rare in Malawi. Both versions of RUTF can also cause illness( abdominal gas, diarrhoea, stomach-ache, malaise) in people with lactose and milk intolerance. Both versions of RUTF could result in kidney dysfunction or damage in Individuals with conditions where high -protein diets could be harmful such as type I diabetes mellitus. Children known to have any of these conditions will not be prescribed any of the RUTFs as part of this study - Taking blood from the finger/arm causes a small amount of pain, bruising, swelling, discomfort, and minimal chance of infection. If an infection should develop, we will provide treatment. We will ensure staff collecting samples are well trained to minimize this risk. The amount taken is too small to affect your child’s health. - **Benefits**. There are no direct benefits to your child for participation in this study. However, your child’s participation will help to understand if proposed high protein quantity and quality in RUTF formulation can improve height or length gain in severely wasted children. If this RUTF formulation improves length and height gain effectively, we expect that future outcomes amongst severely wasted children will be improved. - **Alternatives.** If you choose your child not to participate, your child will continue to be treated with the standard RUTF and any medication related to any present illness at this health facility according to current best guidelines and practices. |

**Introduction:**

**Your child’s illness/symptoms**

Your child has been examined by the doctor/clinician and found to have severe wasting requiring treatment with specially formulated treatment food and medical treatment according to Malawi’s guidelines for treating severe acute malnutrition. Your child will undergo physical examination which is part of usual assessments for all severely wasted children as part the treatment.

**Who is carrying out this study?**

This study is being carried out by the Childhood Acute Illness & Nutrition Network (CHAIN) Malawi group at Kamuzu University of Health Sciences in collaboration with the Hospital for Sick Children, Toronto, Canada and the University of Toronto. In Malawi, CHAIN works in collaboration with the Ministry of Health and Blantyre District Health Office.

**What is this study about?**

- We aim to find out if giving higher protein quantity and quality RUTF can improve length or height gain in severely wasted children and help them reach their age-appropriate length or height.

Severely wasted children admitted at nutrition treatment clinics at health centers will be allocated by chance (randomization) to receive either standard /usual RUTF or higher protein RUTF. Both RUTFs will be given for the period of 8 weeks. The decision on which child gets which RUTF will be decided by a (randomization) system based on chance, without any preference. This means that all participants will have the same chance of being given the usual RUTF or high protein RUTF.

We will find out if there is any difference between usual RUTF and high protein RUTF by closely observing the progress of every child in this study. To make sure the findings of this study are as accurate as possible, it is important that no one, including you and the doctors and researchers caring for your child, knows which child is receiving which RUTF until the end of the study. To ensure that no-one knows which type of RUTF a particular child is receiving, each type of RUTF will be assigned to a unique code and either purple packaging (RUTF-99) or grey packaging (RUTF-88). The colors will be unique to the type of RUTF. This blinding of the RUTFs will help to prevent everyone involved in the study from favoring either type of the RUTF in the research conduct study

- This study will take place in Blantyre district, at Limbe, Mbayani Ndirande and Bangwe health centres.
- We aim to recruit 120 children with severe wasting
- We are asking your permission for you/your child to participate in this study.

**What will it involve for me/my child?**

If you agree to have your child to participate in this study:

- Priority will be given to your child to receiving the medical care that maybe needed according to the attending doctor/clinician and health centre’s guidelines.
- The study team will collect information on clinical history and assessments of your child.
- A small amount of blood sample will be obtained from your child when they enroll in the study and on week 4.
- Your child will receive either high protein RUTF or usual RUTF. RUTF is a proper and normal treatment food used in severely malnourished children per Malawi treatment guidelines.

**If you agree to allow your child to participate in this study, we will:**

- **Study medication**: We will assign your child to receive either usual RUTF or high protein RUTF. This will be taken for a period of 8 weeks.
- **Sample collection**: We will take small blood samples (about teaspoon each time) two times during the study (enrollment, week 4) by placing a temporary needle in a vein.
- **Data collection:** During the study we will collect information on how sick or well your child is and how he/she is growing at every week for 8 weeks. We will also ask you questions on your thoughts regarding RUTF use in one interview. We will also ask you if we could visit your home once within two weeks of joining the study to observe how the child eats RUTF at home.
- **Body size/growth assessments:** We will measure your child’s weight, the thickness of your child’s mid upper arm/mid-upper arm circumference (MUAC) as well as length/height at every clinic visit. We will also measure, the length of your child’s lower leg (knee-heel length), and size of your child, skin pinch (skin fold thickness at admission), and week 0 and week eight.
- **Body composition:** We will measure the proportion of body fat and non-fat in your body using low electric current using a method called bioelectrical impedance assessment

**Are there any risks or disadvantages to me/my child of taking part?**

- Our priority for every participant is their well-being.

These RUTFs are almost the same in everything except the protein quantity and quality through the addition of more dairy. Therefore, there is no risk or very low risk if any, but we will still be monitoring your child closely. If any new information about the high protein RUTF becomes available during this study, we will let you know. Currently, it is known that both the standard RUTF and high protein RUTF can cause allergic reaction in people with peanut allergies. Both versions of RUTF can also cause illness( abdominal gas, diarrhoea, stomach-ache, malaise) in people with lactose and milk intolerance. Both versions of RUTF could also result in kidney dysfunction or damage in Individuals with conditions where high -protein diets could be harmful such as type I diabetes mellitus. Children known to have any of these conditions will not be prescribed any of the RUTFs as part of this study

- Taking blood from the finger/arm causes a small amount of pain, bruising, swelling, discomfort, and minimal chance of infection. If this happens, we will provide treatment. We will ensure staff collecting samples are well trained to minimize this risk. The amount taken is too small to affect your child’s health.
- You will be asked to bring your child back for follow up at this clinic. Follow up visits will take some of your time, estimated at about 1 hour at the clinic in addition to your travel time to the clinic and back. We will however reimburse transport costs you incur to the clinic and back for every visit and a pocket allowance of MWK 5000 at week 4 and week 8 follow ups.
- An independent committee will monitor this research continuously to ensure the safety and rights of all research participants are always respected.
- To ensure safety of you and your child during the study against infections including Covid-19, we will promote social distancing and other safe practices during ward care and follow up according to ministry of health guidelines.

**What happens if I refuse to participate?**

All participation in research is voluntary. You are free to decide if you want/your child wants to take part. Your child will still receive the recommended standard of care for malnutrition and illness they have even if they do not take part. If you do agree to participate, you can change your mind at any time and withdraw your child from the research. Previously collected information and samples will be withdrawn and not used in the analysis, at your request This will not affect you/your child’s care now or in the future.

**What happens to the samples?**

- Individual names will be removed from all samples and replaced by codes, to ensure that samples can only be linked to the participants by people closely concerned with the research.
- All the samples will be tested and analyzed here in Malawi at the Kamuzu University of Health Sciences, South Africa, or Canada.
- There will be no development of products of commercial importance from specimens used in this research.

**Who will have access to information about me/my child in this research?**

All our research records will be stored securely in locked cabinets and password-protected computers. Only the people who are closely concerned with the research will be able to view information from participants, in order to be sure that the study is being run correctly and the privacy of every participant is protected. These individuals will keep the information confidential.

In the future, information collected or generated during this study may be used to support the development of new policy/guidelines on the management of severe wasting. This study’s findings may also be used to support other research by other researchers. In all cases, we will only share information with other researchers in ways that do not reveal individual participants’ identities. For example, we will remove information that could identify people, such as their names and where they live, and replace this information with number codes. Any future research using information from this study must first be approved by a local or national expert committee to make sure that the interests of participants and their communities are protected.

**What if the researchers discover something about my child?**

The blood tests being done as part of this study are designed to answer research questions, not to examine your child’s blood for medical purposes. This research blood test is not a substitute for one that a doctor would order, and it may not show problems that would be picked up by a clinical blood test. A research staff will review your child’s blood test results acquired as part of this research study. In the very unlikely event that abnormal findings are found, the Study Doctor will contact your child’s treating doctor about any findings that may affect your child’s car

**Who has approved this research?**

Most clinical research in Malawi must be approved before they begin by local and international committees, which includes National Health Sciences Research Committee (NHRSC). They must agree that the research is important, relevant to country of research and follows nationally and internationally agreed research guidelines. This includes ensuring that all participants’ safety and rights are respected.

What if I have any questions?

*You are free to ask questions of any staff at any time. You can also contact the research team using these contacts:*

| **Chisomo Eneya**  **Study Coordinator**  **Childhood Acute Illness Nutrition Network,**  **Kamuzu University Health Sciences**  **Malawi, 0999 20 94 81** | **Isabel Potani,**  **PhD Candidate, CO-Principal Investigator**  **University of Toronto (Department of Nutritional Sciences), Canada, and Kamuzu University Health Sciences**  *,* **0992319586.** |
| --- | --- |

*If you want to ask someone independent about this research, please contact:*

**The Chairman,**

**National Health Sciences Research Committee,**

**Ministry of Health (Research Department,**

**P.O. BOX, 30O377,**

**Lilongwe 3,**

**Malawi**

**Protein + trial: Patient information and consent form**

**Study title: A proof-of-concept randomized control trial on the role of higher protein quantity and quality-ready-to-use therapeutic food in improve linear growth among 6–23-month-old children with severe wasting. “The Protein + trial”**

**LAY TITLE*: Improving height and length in children using high protein RUTF***

I, being a parent/guardian of ________________________________ (name of child), have had the research explained to me. I have understood all that has been read/explained and had my questions answered satisfactorily and I agree to allow my child to take part in the research.

I understand that allowing my child to take part in this research may include shipment of my child’s samples abroad to Canada and South Africa.

| **I agree to my/my child’s samples being stored and used for the current study on treatment of severe wasting** | **Yes ÿ No ÿ** |
| --- | --- |
| **I agree to my/my child’s samples being exported for further analyses as described** | **Yes ÿ No ÿ** |
| **I agree to my/my child’s samples being stored and used for future studies on treatment of severe wasting** | **Yes ÿ No ÿ** |
| **I agree to my/my child’s samples being exported for further analyses including future studies** |  |
| **I agree to a home visit by the research team to observe RUTF intake** | **Yes ÿ No ÿ** |

I understand that I can change my mind at any stage, and it will not affect me or my child in any way.

**Participant/Parent/guardian’s signature**: ____________________________**Date** ___________

**Participant/Parent/guardian’s name:** _____________________________ **Time** ____________

(Please print name)

------------------------------------------------------------------------------------------------------------------------------------------

***Where participant/parent/guardian cannot read***

I attest that the information concerning this research was accurately explained to and apparently understood by the participant/parent/guardian and that informed consent was freely given by the participant/parent/guardian.

**Witness’ signature*:** _____________________________________ **Date** _____________

**Witness’ name***: _____________________________________ **Time** ______________

(Please print name)

**A witness is a person who is independent from the study or a member of staff who was not involved in gaining the consent.*

Thumbprint of the participant/parent/guardian as named above if they cannot write:

------------------------------------------------------------------------------------------------------------------------------------------

- I have followed the ethical procedure to obtain consent from the [participant/parent/guardian]. S/he apparently understood the nature and the purpose of the study and consents to the participation [of the child] in the study. S/he has been given opportunity to ask questions which have been answered satisfactorily.

**Designee/investigator’s signature:** ____________________________ **Date** ____________

**Designee/investigator’s name:**  _____________________________**Time** ____________

(Please print name)

**THE PARTICIPANT/PARENT/GUARDIAN SHOULD NOW BE GIVEN A SIGNED COPY TO KEEP**
