# Supplementary material for: A protocol for a proof-of-concept randomized control trial testing increased protein quantity and quality in ready-to-use therapeutic food in improving linear growth among 6-23-month-old children with severe wasting in Malawi
Source: PLoS One. 2023 Aug 24;18(8):e0287680. doi: 10.1371/journal.pone.0287680 (PMC10449476; doi:10.1371/journal.pone.0287680)
Supplement: S2 File — (DOCX) [file pone.0287680.s005.docx]

**A protocol for proof-of-concept randomised control trial on the role of higher protein quantity and quality-ready-to-use therapeutic food in improving linear growth among 6-23-month-old children with severe wasting. “The Protein + trial”**

1. **INVESTIGATORS**

|  | |
| --- | --- |
| **Principal Investigator** | |
| Dr. Robert Bandsma (Supervisor) | Associate Professor of Paediatrics  The Hospital for Sick Children  Canada |
| **Co-Principal Investigator** | |
| Ms. Isabel Potani | PhD Candidate  University of Toronto (Department of Nutritional Sciences), Canada,  Canada |
| **Co-Investigators** | |
| Dr. Emmie Mbale (Supervisor) | MBBS, MMed  Queen Elizabeth Central Hospital  Blantyre  Malawi |
| Dr. Allison Daniel | Independent nutrition consultant  Canada |
| Dr Glenda Courtney-Martin | Assistant Professor  The Hospital for Sick Children  Department of Nutritional Sciences  Canada |
| Laura Vresk | Research Fellow & Clinical Dietitian  The Hospital for Sick Children  Canada |
| Dr. André Briend | Adjunct Professor  Tampere University.  Finland |
| Mr. Chisomo Eneya | Study Clinician  Childhood Acute Illness Nutrition Network  Kamuzu University of Health Sciences, Malawi |
| Mr. Sylvester Kathumba | Nutritionist  Department of Nutrition and HIV  Malawi |
| Dr. Wieger Voskuijl | Paediatrician and honorary consultant in Paediatrics and Child Health  Amsterdam Centre for Global Child Health, Emma Children’s Hospital, Amsterdam University Medical Centre  The Netherlands |
| Dr. James Berkley | Professor Of Paediatric Infectious Diseases  Nuffield Department of Medicine  University of Oxford |

1. **ABSTRACT**

**Background and problem**:

RUTF has successfully promoted recovery from severe wasting and widened treatment coverage. However, RUTF does not sufficiently promote linear growth, leaving many survivors of severe wasting at risk of persistent stunting. Stunting is associated with long-term effects like poor child development and a high risk of non-communicable disease in adults. High protein quantity and quality stimulate linear growth; however, an RUTF with a higher protein quantity and quality than the standard RUTF has yet to be tested. We hypothesise that the suboptimal linear growth in children surviving severe malnutrition can be improved by increasing the protein quantity and quality in the standard RUTF formulation. We designed a high protein quantity and quality RUTF that will be tested in a proof-of-concept quadruple-blind randomised controlled trial study design. **Aim/Objective**:

The trial aims to compare the efficacy of an 8-week high-protein-RUTF and standard RUTF in improving linear growth among 6-23 months old children with severe wasting.

**Outcomes**:

The primary outcome in this proof-of-concept study is a change in insulin-like growth factor-1 (IGF- 1) after four weeks of treatment. IGF-1 is a mechanistically linked hormone and positively associated with linear growth. Secondary outcomes are changes from baseline measured at eight weeks for: height, knee-heel length, weight as well as muscle mass deposition, and amino acid profile. These findings are crucial to inform future management, including therapeutic food for treating severe wasting to improve linear growth.

**Constraints:** We will adhere to all local COVID-19 preventative measures to reduce the COVID -19 risk to participants. All analysis will be done in Malawi to avoid shipment challenges due to COVID-19-related shipment challenges.

**Dissemination**: We will publish the results in peer-reviewed journals and share the findings with stakeholders such as the Malawi ministry of health, World Health Organization, NHRSC and Blantyre DHO.

1. ***BACKGROUND* AND LITERATURE REVIEW**

**Severe Wasting**

Severe wasting is diagnosed by weight-for-height z-scores (WHZ) below -3SD or mid-upper arm circumference (MUAC) below 115mm. Severe wasting and oedematous malnutrition are operationally called severe acute malnutrition (SAM) (1). The concurrency of SAM and acute infections or anorexia or (nutritional) oedema is termed complicated SAM. Conversely, SAM in the absence of the mentioned clinical features is termed uncomplicated SAM. The mortality rate related to complicated SAM can reach up to 30% (2–4), while mortality related to uncomplicated SAM reaches up to 10% (5–9).

.The mortality rate related to complicated SAM can reach up to 30% (2–4), while mortality related to uncomplicated SAM reaches up to 10% (5–9).

Children with severe wasting generally also present with stunting (10). Stunting is defined by a height-for-age z score (HAZ) that is less than -2 SD (11). Stunting is associated with poor long-term outcomes, including poor child development, lower school achievement and income, and increased risk of noncommunicable diseases (12–15)**.**

**Outpatient Therapeutic Programs**

Children with medically complicated SAM are first stabilised in inpatient facilities. In the absence or after the stabilization of medical complications, SAM can be successfully managed in outpatient therapeutic feeding programs (OTP) (16). During OTP, children are prescribed oral antibiotics to treat subclinical infections (17) and are provided with RUTF to promote nutritional recovery. Successful treatment of severe wasting is defined by WHZ of at least -3 standard deviations, or MUAC that is at least 115mm and typically takes at least 3-8 weeks (18). Children who fail to recover after eight weeks of treatment are classified as ‘treatment non-responders’ and require further clinical and social investigation (16). ***Importantly, despite completing nutrition rehabilitation with RUTF, severely wasted children often relapse and remain stunted*** (19).

RUTF was developed in 1996 for children with SAM based on the composition of another WHO-recommended therapeutic feed, F-100(20,21), and has been highly effective in reducing mortality in children with severe wasting (18,22,23). RUTF is made of ingredients embedded in a lipid-rich paste, resulting in an energy-dense food with low water activity, thus, has a low risk for microbial activity (24). Typical ingredients include milk powder, vegetable oil, sugar, peanut butter and powdered vitamins and minerals, but the composition may vary locally; thus, different versions exist (25). As the name implies, RUTF does not need any preparation before consumption, making it practical in resource-limited settings where cooking fuel and facilities are limiting constraints (24,25).

**Role of protein quality and quantity in growth stimulation**

Although poor micronutrient status (26,27), systemic inflammation and enteric dysfunction (28) have been associated with poor linear growth, there is also strong evidence indicating that poor dietary protein quality and quantity is associated with stunting (29–31). This is essential in SAM management as protein requirements are higher during SAM recovery than during normal growth periods due to catch-up growth (32). In addition to inadequate protein intake, reduced digestive pancreatic enzyme secretion and intestinal epithelial dysfunction contribute to inadequate protein availability in children with severe wasting (26,27). However, high protein intake has been associated with mortality in individuals with, e.g., unresolved complicated SAM. Although there is no direct evidence, it is believed that high protein diets complicated SAM is associated with mortality due to compromised liver function in SAM (27,28)

There is sufficient evidence in children with severe wasting (uncomplicated SAM) to suggest that increasing total protein quantity and protein quality in RUTF could safely provide adequate amino acids for the restoration of normal physiological processes necessary to promote linear growth(28). However current ***standard RUTF formulations do not meet the minimum protein quality score set by the Food Agricultural Organization (FAO).*** ***We, therefore, hypothesise that a higher protein quantity and quality of RUTF is required to improve linear growth and related outcomes in 6–23-month-old children recovering from severe wasting.*** The Protein Digestibility Corrected Amino Acid Score (PDCAAS) of standard RUTF is 0.83, while the minimum PDCAAS set by FAO is at least 1 (29,30). The protein quantity and quality of RUTF could be improved by adding whey, a dairy product with high protein quantity and quality but is cheaper than skimmed milk (31,32).

**Mechanism of action of protein on linear and ponderal growth**

As shown in figure 1, sufficient protein intake directly activates the main growth regulator, the Mechanistic Target Of Rapamycin Complex 1 (MTORC1). Also, it modulates growth by up-regulating growth factors such as growth hormone, IGF-1 and insulin-like growth binding protein 3 (IGFBP3) (21,33,34). IGF-1 is a growth hormone released by the liver and muscles and promotes MTORC1 activity. IGF-1 travels in the bloodstream bound to IGFBP3. Activation of MTORC1 results in increased cartilage deposition by up-regulating peptide of type I procollagen (PICP), and this leads to bone elongation hence linear growth (35). Sufficient protein intake and MTROC1 interaction also increase skeletal muscle mass deposition. Due to its role in growth, IGF-1 is gaining recognition as a growth marker (36).

**Figure 1: Role of protein quality and quantity in growth modulation**


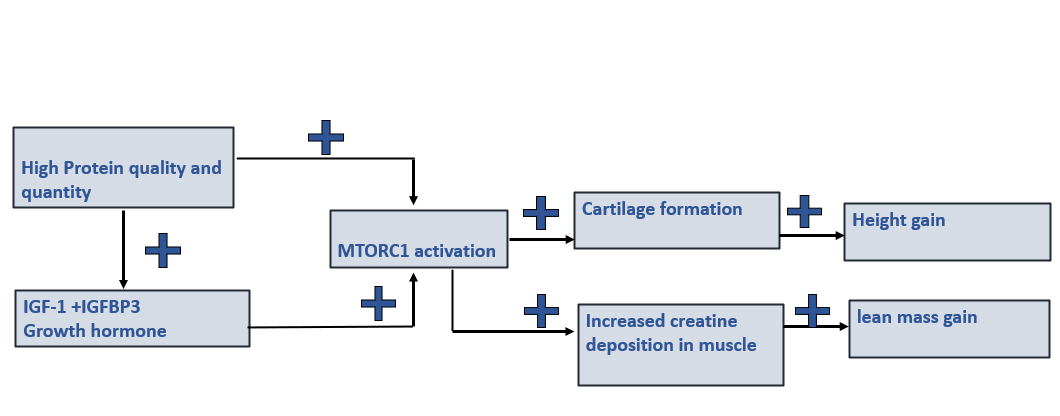


1. **JUSTIFICATION/RATIONALE**

The proposed proof of concept trial explores a novel RUTF formulation aiming at improved height gain, higher recovery rates and lean mass deposition among children with severe wasting. This study can influence policy around the management of children with severe wasting.

1. **OBJECTIVES**
2. **Primary objective**

To assess the efficacy of high-protein-RUTF with higher protein quality and quantity compared to standard RUTF in increasing circulating IGF- 1 after four weeks in 6–23-month-old children with severe wasting.

1. **Secondary objectives**

- To assess the efficacy of high-protein-RUTF (higher protein quality and quantity) compared to standard RUTF in improving:
  - Change in height-for-age z score, weight for height z score, weight for age z score
  - Weight gain per kilogram body weight per day
  - Changes from baseline for each of the outcomes
    - plasma essential amino acids: leucine, threonine and tryptophan
    - skeletal muscle mass using Bioelectrical impedance
    - Knee-heel length (mm)
    - Fat mass and fat-free mass accretion using skin fold thickness
- To assess the acceptability and compliance of the high-protein RUTF formulation compared to standard RUTF among children with severe wasting.
- To assess the safety of high-protein-RUTF compared to standard RUTF.

1. **METHODS**
   - 1. **Trial Design**

A quadruple-blind randomised controlled proof of concept trial will be conducted at two outpatient therapeutic programs (OTP) clinics in Blantyre district, Malawi. There will be two study arms, standard RUTF and high-protein RUTF, as the treatment (as shown in Figure 2 below). The two RUTFs will be isocaloric, but the high-protein RUTF recipe will have more proportions of milk powder, whey protein, and vegetable oil. To promote linear growth, the high-protein RUTF will thus contain both a higher protein quantity and as well as quality (as measured by the digestible indispensable amino acid score (DIAAS) compared to the standard RUTF (see table 1). The high-protein-RUTF will be produced at no cost by Nutriset, Malaunay, France, the leading manufacturer of the global supply of RUTF.

*Randomisation*

Each participant will be randomly assigned to either high or standard protein RUTF individually. Sequential study numbers will be computer generated according to blocked randomisation by the site before the study begins to ensure equal random treatment allocation at each location. An independent statistician will do this.

*Blinding of treatment*

The patients, investigators and data analysts will be blinded from the treatment allocation until data analysis is complete; thus, quadruple blinding. The two RUTFs will be packaged in identical 92g sachets. The RUTFs will be as similar as possible in colour, texture, and smell. The two packages/sachets of the two types of RUTF will differ by colour and a number code, either grey-88 or purple-99 (the colour code will be unique to each RUTF type). An independent statistician will do the colour allocation. The quadruple-blind design using unique colour codes will reduce performance bias due to awareness of the applied intervention among the investigators directly involved in supervision, child recruitment, and management and outcome assessment. At the end of data collection, the independent statistician will provide colour identification to the lead investigator for final data analysis.

|  |  | **RUTF PROT +** | **RUTF control** |  | **RUTF PROT +** | **RUTF control** |
| --- | --- | --- | --- | --- | --- | --- |
| **Components** | Unit | **Per sachet(100g)** | **Per sachet(100g)** |  | **Per sachet(92g)** | **Per sachet (92g)** |
| Calories | Kcal | 550 | |  | 506 | |
| Carbohydrates | % | 35 | 43 |  | 32 | 39 |
| Sucrose | % | 17 | |  | 16 | |
| Sucrose energy | % of total energy | 12 | |  | 12 | |
| Proteins | % | 20.3 | 13.8 |  | 19 | 13 |
| **Protein energy** | **% of total energy** | **15** | **10** |  | **15** | **10** |
| **DIAAS/PDCAAS** |  | **1.18/1.19** | **0.76/0.86** |  | **1.18/1.19** | **0.76/0.85** |
| Alanine | % | 0.8 | 0.5 |  | 0.7 | 0.5 |
| Arginine | % | 0.9 | 0.9 |  | 0.9 | 0.9 |
| Aspartic Acid | % | 1.9 | 1.3 |  | 1.7 | 1.2 |
| Cystine | % | 0.4 | 0.2 |  | 0.4 | 0.1 |
| Glutamic Acid | % | 3.9 | 2.7 |  | 3.6 | 2.5 |
| Glycine | % | 0.5 | 0.5 |  | 0.5 | 0.4 |
| Histidine | % | 0.6 | 0.3 |  | 0.6 | 0.3 |
| Isoleucine | % | 1.1 | 0.6 |  | 1.0 | 0.6 |
| Leucine | % | 1.8 | 1.1 |  | 1.7 | 1.0 |
| Lysine | % | 1.6 | 0.8 |  | 1.5 | 0.7 |
| Methionine | % | 0.4 | 0.2 |  | 0.4 | 0.2 |
| Phenylalanine | % | 0.9 | 0.6 |  | 0.8 | 0.6 |
| Proline | % | 1.2 | 0.9 |  | 1.1 | 0.8 |
| Serine | % | 1.0 | 0.7 |  | 0.9 | 0.6 |
| Threonine | % | 1.0 | 0.5 |  | 0.9 | 0.5 |
| Tryptophan | % | 0.3 | 0.2 |  | 0.3 | 0.2 |
| Tyrosine | % | 0.8 | 0.6 |  | 0.8 | 0.5 |
| Valine | % | 1.2 | 0.7 |  | 1.1 | 0.6 |
| Met/Cys |  | 1.0 | 1.5 |  | 1.0 | 1.5 |
| Phe/Tyr |  | 1.0 | 1.5 |  | 1.0 | 1.5 |
| Lipides | % | 36.2 | 35.8 |  | 33.3 | 33.0 |
| Lipides energy | % of total energy | 59 | |  | 59 | |
| Acide linoléïque C18:2 | % | 3.9 | 3.7 |  | 3.6 | 3.4 |
| C18:2 energy | % of total energy | 6.4 | 6.1 |  | 6.4 | 6.1 |
| Acide linolénique C18:3 | % | 0.8 | 1 |  | 0.7 | 0.7 |
| C18:3 energy | % of total energy | 1.3 | |  | 1.3 | |
| Calcium | Mg | 452 | |  | 416 | |
| Copper | Mg | 1.6 | |  | 1.4 | |
| Iron | Mg | 12 | |  | 11 | |
| Iodine | µg | 88 | |  | 81 | |
| Magnesium | Mg | 110 | |  | 101 | |
| Manganese | Mg | 0.2 | 0.3 |  | 0.2 | 0.3 |
| Free phosphorus | Mg | 378 | 429 |  | 348 | 395 |
| Potassium | Mg | 1300 | |  | 1196 | |
| Sodium | Mg | 152 | |  | 140 | |
| Selenium | µg | 30 | |  | 28 | |
| Zinc | Mg | 13 | |  | 12 | |
| Vitamin A | µg | 901 | |  | 829 | |
| Vitamin B1 | Mg | 0.6 | |  | 0.5 | |
| Vitamin B12 | µg | 1.8 | |  | 1.7 | |
| Vitamin B2 | Mg | 1.8 | |  | 1.7 | |
| Niacin | Mg | 5.6 | |  | 5.2 | |
| Pantothenic acid | Mg | 3.4 | |  | 3.1 | |
| Vitamin B6 | Mg | 0.7 | |  | 0.6 | |
| Biotin | µg | 68 | |  | 62 | |
| Folic acid | µg | 227 | |  | 209 | |
| Vitamin C | Mg | 56 | |  | 52 | |
| Vitamin D | µg | 18 | |  | 17 | |
| Vitamin E | mg α TE | 23 | |  | 21 | |
| Vitamin K | µg | 18 | |  | 17 | |

**Table 1 nutritional composition of standard (control and high-protein RUTF (protein +)**

1. **Study setting**

The study will be conducted in the Blantyre district in Malawi. There are 27 OTP clinics in Blantyre that operate community health centres under the supervision of the Blantyre District Health Office (DHO). We will recruit from four health centres in Blantyre DHO with the largest uncomplicated SAM admissions compared to other OTP clinics in Blantyre district (as of December 2021); this will allow for adequate sampling within the study timeline. The health centres are Bangwe, Mbayani, Ndirande and Limbe health centres.

1. **Study population**

This is a trial of children between 6 months to 23 months presenting to OTP clinics with uncomplicated severe wasting.

1. **Study period/ timeline**

The study will recruit for an expected duration of seven months and will have an additional six months for data analysis and dissemination.


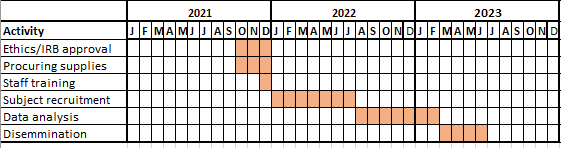


1. **Sample size**

The power was at 80% and the alpha at 0.05. Based on an earlier study, the expected mean difference in change in IGF-1 between the two groups is 39.7 ng/ml with a standard deviation of 66 ng/ml (38), and this yielded 46 children per arm. The sample size was then adjusted for 20% attrition and 10% mortality, resulting in 60 participants per study arm (39). Therefore, the total number of patients needed for this trial is 120 children with uncomplicated SAM.

*Inclusion*

Children aged 6-23 months with severe wasting will be eligible for inclusion if they are newly admitted at OTP on the recruitment day. Only children admitted with severe wasting according to the WHO 2013 severe wasting criteria are eligible: WHZ below -3 or MUAC below 115mm (10). Additionally, for the child to be eligible, the parent or guardian must be able and available to consent. Other eligibility criteria are that the child can feed orally in a normal state of health and that the primary caregiver plans to stay in the study area during the duration of the study.

*Exclusion*

All children with medical complications as defined by WHO 2013 SAM treatment guidelines will be excluded (10). Medical complications include lack of appetite, severe oedematous malnutrition, and acute infections. Additionally, children with cerebral palsy (CP), tuberculosis (TB,) HIV infected or exposed are excluded. Children with a diagnosed terminal illness, e.g., cancer, will also be excluded. Children who had SAM in the last eight weeks, i.e., SAM relapses in the previous eight weeks, and children admitted to any NRU due to complicated SAM in the previous four weeks will also be excluded. These elements of the exclusion criteria were determined to exclude any medically complicated children considering that this is an efficacy proof-of-concept trial. According to the Malawi treatment guidelines, all children with medical complications will be referred for medical treatment.

Other exclusion criteria include children previously enrolled in this trial or children with a sibling presently enrolled in the study. Children with known intolerance or allergy to high protein diets and milk/lactose are also not eligible. Lastly, children whose caregivers refuse to give consent are also not eligible.

*Withdrawal criteria*

Reasons for withdrawal will be recorded in the case report form (CRF):

•Subject decision to withdraw from the study. This is allowed without prejudice or affecting clinical care.

•Any clinically relevant signs, symptoms, or adverse events that, in the opinion of the site Principal Investigator, warrant subject withdrawal for the safety of the participant

•If the subject was enrolled in violation of the study protocol (without unblinding)

•Loss to follow-up (defined as a subject who consistently does not return for protocol study visits, is not reachable by telephone or any other means of communication and/or is not able to be located).

• On the advice of the data safety and monitoring board (DSMB)

*Managing withdrawals*

Every effort will be made to provide information to parents/guardians and treat families respectfully. Participants choosing to withdraw will be referred to any services indicated by the child’s medical and nutritional condition. They will also be invited to seek medical attention at the study clinic for any new symptom that develops until 60 days after enrolment so as not to be disadvantaged in any way from withdrawal.

Data collected on the subject to the point of withdrawal will remain part of the study database. They will not be removed unless parents/guardians specifically request removal and will be included in final analyses. Samples collected until withdrawal from the study will be used for planned analysis unless parents/guardians request that samples be destroyed without analyses.

*Replacing withdrawn participants*

Participants who withdraw will not be replaced. Measures will be put in place at sites to minimise loss to follow-up.

*Informed consent procedure*

Consent will be sought from the caretaker of each child before enrolment. Children seeking treatment at any participating OTP will be screened for eligibility by study field workers at the OTPs. Once their eligibility for treatment is confirmed, a research officer will ensure that the present caretaker is a primary guardian of the child. If yes, the research officer will verbally explain the details of the study to the caregiver in the local language as written in the informed consent form, as there are significantly low levels of adult literacy in this population of interest. Caregivers will nonetheless be provided time to read the consent form before signing in, providing information and administering the consent procedure. Where a caregiver cannot read or write, an impartial witness will be used to document that consent has been provided. A written signature or thumbprint of the parent/ guardian will mark consent to participation in the study.

Suppose the caretaker is not a primary guardian or chooses not to participate in their child is enrolled for treatment only, their information will not be collected, and there will not be any penalties (i.e., it will not affect their care).

The research team will be trained in Good Clinical Practice and will also be trained on a standard operating procedure for obtaining consent using didactic learning and role plays.

1. **Data Collection**

As shown in figure 2, the main data collection time points for the outcome variables are at enrolment, four weeks, and eight weeks using the weekly record form attached. The weekly record form will also collect routine data as part of OTP at each visit. The study will also collect baseline data on the child’s sex, date of birth, area of residence and socioeconomic status (using the enrolment form attached). Caregivers and their infants will be asked to return to the OTP clinic to obtain RUTF every two weeks (per standard OTP frequency) for a total of 8 weeks of treatment. Participants will be issued a reminder of the follow-up visit by phone call (or text message), and defaulters will be traced at home. If data is not collected on the prespecified time point, data can be collected within seven days of the missed data collection time.

1. **Assessment of outcomes**

Table 2 shows a complete list of the study’s outcomes of interest, namely, anthropometry body composition, biochemical, morbidity, and safety outcomes.

*Anthropometry*

Our research team will conduct all routine anthropometric assessments at every OTP visit, i.e., weight and MUAC, as per the WHO growth assessment protocol. We will also use knee-heel length (42), in addition to full body length as leg length is more responsive to environmental stimulants than full body length (43).

*Biochemical assessments*

We will also collect venous blood samples for IGF-1 IGFBP3 and amino acid profiles (leucine, threonine and tryptophan), which are positively associated with growth (44). Plasma IGF-1 and IGFBP3 will be analysed using a standard enzyme-linked immunosorbent assay. Amino acid profiles will be assessed as an exploratory outcome using liquid chromatography. A 3 ml blood sample will be collected at enrolment and week four; this is in accordance with research regulations in Malawi.

**Figure 2: Flow diagram of study activities at OTP clinics**

- Anthropometric assessments: height, kneel-heel length, weight, MUAC
- Body composition assessment: skeletal and muscle deposition, skin fold thickness, bioelectrical impedance assessment
- Growth modulation: IGF-1, IGFBP3, serum amino acids

- Anthropometric assessments: height kneel-heel length, weight, MUAC
- Body composition assessments: skeletal and muscle deposition, skin fold thickness and bioelectrical impedance assessment

- Anthropometric assessments: weight and height
- Growth modulation: IGF-1, IGFBP3, serum amino acids

**Eligibility Assessment**

**Allocate to standard RUTF for 8 weeks.**

**Allocate to High protein RUTF for 8 weeks.**

**Exclusion**

- Acute infection as per CMAM
- TB, CP, HIV diagnosis
- Oedema
- NRU admission in last 4 weeks or OTP admission in last 8 weeks
- Refused consent.

Randomisation

Follow up: Week 4

Baseline Week 0

Follow up: Week 8

The blood samples will be stored at a lab at KUHeS, College of Medicine campus, using the lab CRF attached.

**Table 2: Outcomes of interest**

| **Primary Outcome** | **Description** |
| --- | --- |
| Change in IGF-1 | 1. Change in IGF-1 from baseline to 4th week follow-up |
| **Secondary outcomes** | **Description** |
| Anthropometry | 1. Change in height- or length-for-age z-score (measures linear growth) 2. Change in height mm/day 3. Weight gain in grams per kg bodyweight per day 4. Change in knee-heel length mm/day 5. Change in Weight-for-age z-score (composite measure of linear and ponderal growth) 6. Change in Weight-for--length z-score (measure of wasting) 7. Mid-upper arm circumference mm/day (measure of wasting) 8. Change in fat free mass (FFM) and fat mass (FM) using skinfold thickness and BIA |
| Other OTP outcomes | 1. Default (percentage of children who are absent for two consecutive visits) 2. Non-responder (percentage of children who have not been cured within 8 weeks) 3. Relapse (percentage of children who re-enrolled in OTP after being cured) 4. Wasting Recovery (percentage of children cured, defined as mid-upper arm circumference >115mm, clinically well at 8 weeks following the intervention 5. Mortality (percentage of children who died) by 8 weeks post admission to OTP/initiation of RUTF |
| Growth modulation mechanism | 1. Change in essential amino acids in serum 2. Change in IGFBP3 3. Change in PICP |
| Compliance | 1. Difference between RUTF ration provided and RUTF sachets returned at follow up |
| Acceptability | 1. Completion of prescribed RUTF as per the ACF appetite test (45) |
| Morbidity | 1. Fever 2. Diarrhea 3. Cough 4. Health care visits |
| Safety: Severe Adverse Events) | 1. All SAEs |

*Body Composition Assessment*

Body composition will be assessed using triceps skinfold thickness (TS) and MUAC. Additionally, single-frequency bioelectrical impedance assessment (BIA) will also be used for body composition assessment. TS and MUAC as well as BIA are accepted body composition tools that are feasible in field settings due to their low cost and are relatively easy to use (46).

TS will be measured using Tanner/Whitehouse callipers (Holtain Ltd, UK)(47). The equations below will be used to estimate body composition (53,55).

1. Total arm area
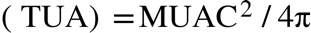

2. Upper arm fat area
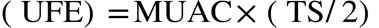

3. Upper arm muscle area estimate
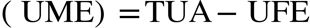

4. % of fat in the upper arm area=
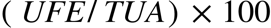


BIA will be measured using a Bioelectrical Impedance Analyzer (BodystatQuadScan4000) at 50 kHz as per the manufacturer's instructions and as was done in a similar study among children with SAM (48).

Acceptability

To assess acceptability, appetite tests will be conducted at every weekly visit based on a protocol designed by Action Contre la Faim and WHO, as shown in Table 3 (48).

**Table 3: Appetite test**

| Steps in conducting an appetite test | |
| --- | --- |
| 1. The appetite test should be conducted in a separate quiet area.  2. Explain to the caregiver the purpose of the appetite test. 3. Explain how it will be carried out.  4. The caregiver should wash her hands.  5. The caregiver should sit comfortably with the child on his lap.  6. She should offer the RUTF from the packet or put a small amount on her finger and give it to the child.  7. The caregiver should offer the child the RUTF gently, encouraging all the time. If the child refuses, then the caregiver should continue to quietly encourage the child. She can take time for the test. The child must not be forced to take the RUTF 8. The child needs to be given plenty of water from a cup as he/she is taking the RUTF | |
| Assessment of appetite test outcome | |
| Child weight | Test passed if the child eats this amount, or more |
| Less than 4 kg | 1/8 of the sachet |
| More or equal than 4 kg, but less than 7 kg | 1/4 of the sachet |
| More or equal than 7 kg, but less than 10 kg | 1/3 of the sachet |
| More or equal than 10 kg, but less than 15 kg | 1/2 of the sachet |
| More or equal than 15 kg, but less than 30 kg | 3/4 of the sachet |
| More or equal than 30 kg | 1/1 of the sachet |

Additionally, to assess acceptability, observed intake of the RUTFs at home will be conducted after two weeks of recruitment. The two-week post-recruitment time point was selected as rapid weight gain is expected within the first two weeks of treatment. The observers will assess how much RUTF is given between 8 am and 12 pm and if this corresponds with the prescribed RUTF. The fieldworker will also observe the number of leftovers as well as the management of leftovers. After observing the intake at any given feed within the observation time window, a questionnaire will be administered by a field worker to evaluate the reaction as perceived by the caretaker and the caretaker's own perception, based on a 5-point hedonic scale, where 1=dislike very much, 2=dislike, 3=neither like nor a dislike, 4=like and 5=like very much. The scale will be illustrated in a series of human face symbols with varying degrees of smile or discontent, which is a method previously used to measure the acceptability of similar treatment foods in a similar population(55). Weight gain will also be interpreted as a proxy measure of acceptability.

Compliance

On the same day of direct observation of RUTF intake at home, a study fieldworker will conduct in-depth interviews with the caregiver record. A semi-structured guide will be used to assess the caregiver's knowledge of the role of RUTF and the use of RUTF in the household. Specific areas of interest in RUTF are the quantity and frequency of providing RUTF to the child, sharing within and outside households, selling of RUTF and any losses of RUTF at home.

At each visit, caregivers will be also asked to bring back empty and full sachets of RUTF to measure compliance.

1. **Safety**

We consider this nutrition intervention-based trial very low risk given that this is a low-risk population. The intervention, high-protein-RUTF, is considered very safe because its formulation is based on the control, the WHO-approved RUTF formulation. The higher protein RUTF only differs from standard RUTF in protein quantity and quality, i.e., moderately increased protein quantity and moderately improved quality.

Despite the minimal safety concerns, each patient will be monitored for clinical evidence of adverse events (AEs) throughout the follow-up period (Table 4). The adverse events of interest are mainly morbidities( including signs of peanut allergies) and mortality occurring during treatment. At every study visit, the research nurse will perform a routine physical examination on the children to assess morbidity and will also ask the caregiver if the child was ill in the previous week to assess any morbidity that may have occurred but has resolved. Attempts to contact children lost to follow-up will be made to assess the mortality status. The cause of death for all deaths occurring within the study time frame will be assessed and recorded using a verbal autopsy form. Children who fall ill and require inpatient care will be referred to inpatient treatment facilities as per local treatment guidelines and will be considered to have reached the study endpoint.

Although peanut allergies will be evaluated as part of safety monitoring as per table 4, we do not anticipate peanut allergies with either product as peanut allergies are low in this population (58,59). Furthermore, the standard RUTF is used in this population with less than 1 % incidence of peanut allergy. Other adverse events of interest will be morbidity, as listed in table 2.

Using the adverse event log attached, details of a reported adverse event will be collected. The information collected will include the date of onset, description, severity, time course, duration and outcome, the relationship of the AE to study medication, concomitant interventions and any action(s) taken. AEs will also be recorded if observed by site personnel or reported spontaneously by the patient.

**Table 4: Definition and classification of adverse events**

| **Term** | **Definition** |
| --- | --- |
| Adverse Event (AE) | *Any untoward medical occurrence* in a patient or clinical investigation subject occurring in any phase of the clinical study whether considered related to the investigational product. This includes an exacerbation of pre-existing conditions or events, intercurrent illnesses, or drug interactions.  Anticipated day-to-day fluctuations of pre-existing conditions, that do not represent a clinically significant exacerbation, will not be considered AEs. Discrete episodes of chronic conditions occurring during a study period will be reported as adverse events to assess changes in frequency or severity.  Adverse events will be documented in terms of a medical diagnosis(es). When this is not possible, the AE will be documented in terms of signs and symptoms observed by the investigator or reported by the subject.  Pre-existing conditions or signs and/or symptoms (including any which are not recognized at admission but are recognized during the study period) present in a subject prior to the start of the study will be recorded on the medical history form within the subject's CRF. |
| Adverse Reaction (AR) | *An untoward and unintended response* in a participant to an investigational product which is related to any dose administered to that participant.  The phrase "response to an investigational medicinal product" means that a causal relationship between a trial medication and an AE is at least a reasonable possibility, i.e., the relationship cannot be ruled out.  All cases judged by either the reporting medically qualified professional or the Sponsor as having a reasonable suspected causal relationship to the trial medication qualify as adverse reactions. It is important to note that this is entirely separate to the known side effects. *It is specifically a temporal relationship between taking the drug, the half-life, and the time of the event or any valid alternative aetiology that would explain the event*. |
| Serious Adverse Event (SAE) | A serious adverse event is any untoward medical occurrence that:   - results in death - is life-threatening. - results in persistent or significant disability/incapacity - rehospitalization for any reason (in a participant who was discharged) or an important medical event leading to severe clinical deterioration requiring medical or surgical intervention to prevent one of the outcomes listed above (i.e., a new diagnosis of TB not requiring admission)   Other ‘important medical events’ may also be considered serious if they jeopardize the participant or require an intervention to prevent one of the above consequences.  **NOTE:** The term "life-threatening" in the definition of "serious" refers to an event in which the participant was at risk of death at the time of the event; it does not refer to an event which *hypothetically* might have caused death if it were more severe. |
| Serious Adverse Reaction (SAR) | An adverse event that is both serious and, in the opinion of the reporting Investigator, believed with reasonable probability to be due to one of the trial treatments |
| Suspected Unexpected Serious Adverse Reaction (SUSAR) | An adverse reaction, the nature or severity of which is not anticipated based on the applicable product information is considered as an unexpected adverse drug reaction. Where the adverse reaction is also considered to have a possible, probable, or definite relationship with the drugs given, and meets the criteria for a serious adverse reaction, it is termed a Suspected Unexpected Serious Adverse Reaction (SUSAR). These events are subject to expedited reporting as for SAEs. |

NB: to avoid confusion or misunderstanding of the difference between the terms “serious” and note of clarification is provided: “Severe” is often used to describe intensity of a specific event, which may be of relatively minor medical significance. “Seriousness” is the regulatory definition supplied above. Detailed guidance can be found here: http://ec.europa.eu/health/files/eudralex/vol-10/2011

**Table 5 Grading of food-induced anaphylaxis according to severity of clinical symptoms**

| **Grade** | **Skin** | **Gastrointestinal** | **Respiratory** | **Cardiovascular** | **Neurological** |
| --- | --- | --- | --- | --- | --- |
| 1 | Localized pruritus, flushing, urticaria, angioedema | Oral pruritis, mild lip swelling | None | None | None |
| 2 | Generalized pruritus, flushing, urticaria, angioedema | Any of the above, Nausea and or emesis x1 | Nasal congestion and/or sneezing | None | Change in activity level |
| 3 | Any of the above | Any of the above plus repetitive vomiting | Rhinorrhoea, marked congestion, sensation of throat pruritus of tightness | Tachycardia | Change in activity level plus anxiety |
|  | Any of the above | Any of the above plus Diarrhea | Rhinorrhoea, marked congestion, sensation of throat pruritus of tightness | Tachycardia | Change in activity level plus anxiety |
| 4 | Any of the above | Any of the above plus Diarrhea | Any of the above, hoarseness, “barky “cough, difficulty swallowing, dyspnoea, wheezing, cyanosis | Any of the above, dysrhythmia and or mild hypotension | “light headedness”, feeling of “pending doom” |
| 5 | Any of the above | Any of the above, loss of bowel control | Any of the above, respiratory arrest | Severe bradycardia and/or hypotension or cardiac arrest | Loss of consciousness |

**Severity Assessment (monitoring)**

Adverse events will be graded according to the Division of AIDS (DAIDS) as shown in Table 6

**Table 6: Grading of adverse events**

| **Grade 1** Mild | Asymptomatic or mild symptoms; clinical or diagnostic observations only; intervention not indicated. |
| --- | --- |
| **Grade 2** Moderate | Minimal, local, or non-invasive intervention indicated; limiting age-appropriate instrumental activities of daily living (ADL). |
| **Grade 3** Severe or medically significant but not immediately life-threatening | Hospitalization or prolongation of hospitalization; indicated disabling limiting self-care ADL. |
| **Grade 4** Life-threatening consequences | Urgent intervention indicated. |
| **Grade 5** Death related to AE. | Death |

**Relationship to Investigational Product**

An assessment of the relationship of the event to the investigation product will be undertaken by the co-PI in consultation with the trial coordination team within 24 hours after the identification of an adverse event. This interpretation will be based on the type of event, the relationship of the event to the time of administration, and the known biology of the intervention (**Table 7**).

**Table 7. Guidelines for assessing the relationship of drug administration to an adverse event**

| **0** | **No Relationship** | No temporal relationship to drug ***and*** alternate aetiology (clinical state, environmental or other interventions); ***and*** does not follow known pattern of response to study product |
| --- | --- | --- |
| **1** | **Unlikely** | Unlikely temporal relationship to drug ***and*** *a*lternate aetiology likely (clinical state, environmental or other interventions) ***and*** *d*oes not follow known typical or plausible pattern of response to drug. |
| **2** | **Possible** | Reasonable temporal relationship to drug; ***or*** event not readily produced by clinical state, environmental or other interventions; ***or s***imilar pattern of response to that seen with other drugs |
| **3** | **Probable** | Reasonable temporal relationship to drug; ***and*** event not readily produced by clinical state, environment, or other interventions ***or*** known pattern of response seen with other drugs |
| **4** | **Definite** | Reasonable temporal relationship to drug; ***and*** event not readily produced by clinical state, environment, or other interventions; ***and*** known pattern of response seen with other drugs |

Reporting Serious Adverse Events and/or Toxicity

For each participant, SAEs will be recorded on the attached SAE form. A summary of all SAEs and suspected toxicity events will be reported every three months or as required by local ethical review boards. The report will include the site; study number; date of the event; subject details (initials, sex, and age); nature of the event; relevant history; outcome and a judgement of causality. A summary report of all SAEs will be sent to the Trial Safety Committee, the ethical and regulatory committees and the sponsor.

SAEs deemed causally related to the investigational product, and SUSARs will be initially reported to the sponsor, DSMB and regulatory bodies within 72 hours of the investigators becoming aware of the event (weekends excluded), with a follow-up report being provided within a further eight calendar days.

**Emergency Procedures**

***Equipment and Drugs***

Resuscitation and emergency care equipment and drugs are available at all sites.

***Breaking the Blind***

Emergency unblinding may be undertaken where this will influence the clinical treatment of the participant. Unblinding will not be undertaken to determine if a child should continue receiving the investigational product or if the investigational product has been stopped, and knowledge of the allocation would not affect other treatments.

***Follow-up of Participants***

The investigator will follow up on serious Adverse Events until their resolution or stabilisation or until causality is determined unrelated to trial interventions. The outcome will be assessed as follows:

• Recovered/resolved

• Not recovered/not resolved

• Recovering/resolving

• Recovered with sequelae/resolved with sequelae

1. **Trial governance**

The trial will have a Data and Safety Monitoring Board (DSMB) comprising an independent chair, a statistician from the Kamuzu College of Health Sciences, and a medical doctor from the Blantyre district health office. The DSMB will review the study outcomes and adverse events at the mid-point of the trial. The trial will be registered at https://clinicaltrials.gov/.

1. **ETHICAL CONSIDERATIONS**

Ethical approval will be sought from the National Health Sciences Research Committee (NHRSC) in Malawi and the Research Ethics Board at the Hospital for Sick Children in Canada. Written informed consent of primary caregivers of infants with severe wasting will be obtained from caregivers at the child’s admission to OTP using the attached consent form. For participants who cannot read or write, an impartial witness will be used as per the GCP specifications.

The study procedures may require participants to attend more OTP clinics and spend a longer time at the OTP clinics than they would if not enrolled in the study. For this reason, participants will be given an allowance of 5000 Malawi Kwacha (10 CAD) at week 4 and week 8 as well as 1000 Malawi Kwacha transport reimbursement at each study visit based on minimum compensation and reimbursement fees.

Each participant is assigned a study number that is used on all data collection forms to maintain participant anonymity. All paper data collection tools with participant information will be kept under a locked cabinet at the main study office located at Queen Elizabeth central hospital.

1. **POSSIBLE CONSTRAINTS**

**Impact of COVID-19**

The current study timeline has been adjusted in response to the COVID-19 pandemic; recruitment is planned to start in September 2021 due to the COVID-19 pandemic and associated global restrictions. The study will be implemented whilst observing COVID-19 prevention strategies, if still required, in accordance with the Malawi Ministry of Health guidelines which will be incorporated into standard operating procedures for the trial. Furthermore, while COVID-19 incidence and severity have reportedly been low in low-income countries like Malawi, in the event of a surge of cases in Malawi, the PI will consult Malawi health authorities and SickKids for guidance on study continuation.

We plan to conduct all sample analyses in Malawi as part of capacity building for laboratories. Moreover, this will avoid delays due to shipment restrictions related to COVID-19.

**REQUIREMENTS**

**Registering the trial**

The trial will be registered at https://clinicaltrials.gov/.

1. **Training provided for study staff.**

All data collectors will be trained in good clinical practice to promote ethics and will undergo training for all study procedures before the commencement of the trial.

1. **REPORTING, DISSEMINATION AND NOTIFICATION OF RESULTS**

***Publication policy***

The trial will be registered and published in an open-access peer-reviewed journal regardless of positive or negative results to avoid publication bias.

***Disseminating the results***

Results will be shared nationally through presentations at the National Paediatric Association's annual scientific meetings and international scientific meetings. The results will also be published in a peer-reviewed journal. We also share the results with NHRSC.

**REFERENCES**

1. A Joint Statement by the World Health Organization and the United Nations Children’s Fund. WHO child growth standards and the identification of severe acute malnutrition in infants and children [Internet]. Available from: www.who.int/childgrowth/standards

2. Irena AH, Mwambazi M, Mulenga V. Diarrhea is a major killer of children with severe acute malnutrition admitted to inpatient set-up in Lusaka, Zambia. Nutr J. 2011;10(1).

3. Talbert A, Thuo N, Karisa J, Chesaro C, Ohuma E, Ignas J, et al. Diarrhoea complicating severe acute malnutrition in Kenyan children: A prospective descriptive study of risk factors and outcome. PLoS One. 2012 Jun 4;7(6):e38321.

4. Heikens GT, Bunn J, Amadi B, Manary M, Chhagan M, Berkley JA, et al. Case management of HIV-infected severely malnourished children: challenges in the area of highest prevalence. Vol. 371, The Lancet. Elsevier Limited; 2008. p. 1305–7.

5. Ciliberto MA, Sandige H, Ndekha MJ, Ashorn P, Briend A, Ciliberto HM, et al. Comparison of home-based therapy with ready-to-use therapeutic food with standard therapy in the treatment of malnourished Malawian children: A controlled, clinical effectiveness trial. American Journal of Clinical Nutrition. 2005;81(4):864–70.

6. Collins S, Dent N, Binns P, Bahwere P, Sadler K, Hallam A. Management of severe acute malnutrition in children. Vol. 368, Lancet. 2006. p. 1992–2000.

7. Collins S, Sadler K. Outpatient care for severely malnourished children in emergency relief programmes: A retrospective cohort study. Lancet. 2002 Dec 7;360(9348):1824–30.

8. Linneman Z, Matilsky D, Ndekha M, Manary MJ, Maleta K, Manary MJ. A large-scale operational study of home-based therapy with ready-to-use therapeutic food in childhood malnutrition in Malawi. Matern Child Nutr. 2007 Jul;3(3):206–15.

9. Burza S, Mahajan R, Marino E, Sunyoto T, Shandilya C, Tabrez M. Community-based management of severe acute malnutrition in India: new evidence from Bihar. Am J Clin Nutr. 2015;101(4).

10. Bhutta ZA, Berkley JA, Bandsma RHJ, Kerac M, Trehan I, Briend A. Severe childhood malnutrition. Vol. 3, Nature reviews. Disease primers. 2017. p. 17067.

11. WHO Expert Committe on Physcal Status. The Use and Interpretation of anthropometry. WHO techinical report series. 1995.

12. Victora CG, De Onis M, Hallal PC, Blössner M, Shrimpton R. Worldwide timing of growth faltering: Revisiting implications for interventions. Pediatrics. 2010 Mar;125(3).

13. Bossavie L, Alderman H, Giles J, Mete C. The Effect of Height on Earnings: Is Stature Just a Proxy for Cognitive and Non-Cognitive Skills? The World Bank; 2017. (Policy Research Working Papers).

14. Qureshy LF, Alderman H, Rokx C, Pinto R, Wai-Poi M, Tandon A. Positive returns: cost-benefit analysis of a stunting intervention in Indonesia. J Dev Effect. 2013;5(4):447–65.

15. Guerrant RL, Deboer MD, Moore SR, Scharf RJ, Lima AAM. The impoverished gut - A triple burden of diarrhoea, stunting and chronic disease. Vol. 10, Nature Reviews Gastroenterology and Hepatology. Nature Publishing Group; 2013. p. 220–9.

16. World Health Organization. WHO guideline: updates on the management of severe acute malnutrition in infants and children. World Health Organization; 2013.

17. Jones KDJ, Berkley JA. Severe acute malnutrition and infection. Paediatr Int Child Health. 2014 Dec 1;34 Suppl 1:S1–29.

18. WHO. Community-based management of severe acute malnutrition: A Joint Statement by the World Health Organization, the World Food Programme, the United Nations System Standing Committee on Nutrition and the United Nations Children’s Fund. 2007.

19. Ngari MM, Iversen PO, Thitiri J, Mwalekwa L, Timbwa M, Fegan GW, et al. Linear growth following complicated severe malnutrition: 1-year follow-up cohort of Kenyan children. Arch Dis Child. 2019 Mar 1;104(3):229–35.

20. UNDP/IAPSO -Copenhagen D. Emergency relief items : compendium of generic specificationsNo Title. Copenhagen, Denmark; 1995.

21. World Health Organization. MANAGEMENT OF SEVERE MALNUTRITION: A MANUAL FOR PHYSICIANS AND OTHER SENIOR HEALTH WORKERS. Geneva; 1999.

22. Lenters LM, Wazny K, Webb P, Ahmed T, Bhutta ZA. Treatment of severe and moderate acute malnutrition in low- and middle-income settings: A systematic review, meta-analysis and Delphi process. Vol. 13, BMC Public Health. 2013. p. S23.

23. Manary MJ, Ndkeha MJ, Ashorn P, Maleta K, Briend A. Home based therapy for severe malnutrition with ready-to-use food. Arch Dis Child. 2004 Jun;89(6):557–61.

24. Briend A. Possible use of spreads as a FOODlet for improving the diets of infants and young children. Food Nutr Bull. 2002;23(3):239–43.

25. Manary M. Local production and provision of ready-to-use therapeutic food (RUTF) spread for the treatment of severe childhood malnutrition. Food Nutr Bull. 2006;27(SUPPL.3).

26. Black RE, Allen LH, Bhutta ZA, Caulfield LE, de Onis M, Ezzati M, et al. Maternal and child undernutrition: global and regional exposures and health consequences. Lancet. 2008 Jan 19;371(9608):243–60.

27. Rivera JA, Hotz C, Gonzá Lez-Cossío T, Neufeld L, García-Guerra A. Animal Source Foods to Improve Micronutrient Nutrition and Human Function in Developing Countries The Effect of Micronutrient Deficiencies on Child Growth: A Review of Results from Community-Based Supplementation Trials 1. Vol. 133, J. Nutr. 2003.

28. Morseth MS, Henjum S, Schwinger C, Strand TA, Shrestha SK, Shrestha B, et al. Environmental Enteropathy, Micronutrient Adequacy, and Length Velocity in Nepalese Children: the MAL-ED Birth Cohort Study. J Pediatr Gastroenterol Nutr. 2018 Aug 1;67(2):242–9.

29. Semba RD, Trehan I, Gonzalez-Freire M, Kraemer K, Moaddel R, Ordiz MI, et al. Perspective: The Potential Role of Essential Amino Acids and the Mechanistic Target of Rapamycin Complex 1 (mTORC1) Pathway in the Pathogenesis of Child Stunting. Advances in Nutrition. 2016 Sep 1;7(5):853–65.

30. Semba RD. The rise and fall of protein malnutrition in global health. Vol. 69, Annals of Nutrition and Metabolism. S. Karger AG; 2016. p. 79–88.

31. Manary M, Callaghan M, Singh L, Briend A. Protein Quality and Growth in Malnourished Children. Food Nutr Bull. 2016;37(Supplement 1):S29–36.

32. Pencharz PB. Protein and energy requirements for ‘optimal’ catch-up growth. Vol. 64, European Journal of Clinical Nutrition. Nature Publishing Group; 2010. p. S5–7.

33. Bartels RH, Meyer SL, Stehmann TA, Bourdon C, Bandsma RHJ, Voskuijl WP. Both Exocrine Pancreatic Insufficiency and Signs of Pancreatic Inflammation Are Prevalent in Children with Complicated Severe Acute Malnutrition: An Observational Study. Journal of Pediatrics. 2016 Jul 1;174:165–70.

34. Farràs M, Chandwe K, Mayneris-Perxachs J, Amadi B, Louis-Auguste J, Besa E, et al. Characterizing the metabolic phenotype of intestinal villus blunting in Zambian children with severe acute malnutrition and persistent diarrhea. Azman AS, editor. PLoS One. 2018 Mar 2;13(3):e0192092.

35. Collins S, Myatt M, Golden B. Dietary treatment of severe malnutrition in adults. Am J Clin Nutr. 1998;68(1):193–9.

36. Golden MHN. The development of concepts of malnutrition. J Nutr. 2002;132(7):2117S-2122S.

37. Manary MJ, Yarasheski KE, Berger R, Abrams ET, Hart CA, Broadhead RL. Whole-body leucine kinetics and the acute phase response during acute infection in marasmic Malawian children. Pediatr Res. 2004 Jun;55(6):940–6.

38. FAO Expert Consultation. Dietary protein quality evaluation in human nutrition. Food and Agriculrure Organization of the United Nations. 2011. 1–79 p.

39. Shivakumar N, Jackson AA, Courtney-Martin G, Elango R, Ghosh S, Hodgkinson S, et al. Protein Quality Assessment of Follow-up Formula for Young Children and Ready-to-Use Therapeutic Foods: Recommendations by the FAO Expert Working Group in 2017. J Nutr. 2020;150(2):195–201.

40. Mathai JK, Liu Y, Stein HH. Values for digestible indispensable amino acid scores (DIAAS) for some dairy and plant proteins may better describe protein quality than values calculated using the concept for protein digestibility-corrected amino acid scores (PDCAAS). British Journal of Nutrition. 2017;117(4):490–9.

41. Potani I, Spiegel-Feld C, Brixi G, Bendabenda J, Siegfried N, Bandsma RHJ, et al. Ready-to-Use Therapeutic Food (RUTF) Containing Low or No Dairy Compared to Standard RUTF for Children with Severe Acute Malnutrition: A Systematic Review and Meta-Analysis. Advances in Nutrition: An International Review Journal. 2021 Apr 10;

42. Jousse C, Bruhat A, Ferrara M, Fafournoux P. Physiological concentration of amino acids regulates insulin-like-growth-factor-binding protein 1 expression. Biochemical Journal. 1998 Aug 15;334(1):147–53.

43. Thissen JP, Pucilowska JB, Underwood LE. Differential regulation of insulin-like growth factor I (IGF-I) and IGF Binding Protein-1 messenger ribonucleic acids by amino acid availability and growth hormone in rat hepatocyte primary culture. Endocrinology. 1994;134(3):1570–6.

44. RISTELI L. The carboxyterminal propeptide of procollagen type I (PICP) in serum and biological fluids. Scandinavian journal of clinical & laboratory investigation. Supplement. 1990.

45. Grenov B, Briend A, Sangild PT, Thymann T, Rytter MH, Hother AL, et al. Undernourished Children and Milk Lactose: http://dx.doi.org/101177/0379572116629024. 2016;37(1):85–99.

46. Hoppe C, Mølgaard C, Juul A, Michaelsen KF. High intakes of skimmed milk, but not meat, increase serum IGF-I and IGFBP-3 in eight-year-old boys. Eur J Clin Nutr [Internet]. 2004 Sep [cited 2021 Jan 29];58(9):1211–6. Available from: https://pubmed.ncbi.nlm.nih.gov/15054433/

47. Linneman Z, Matilsky D, Ndekha M, Manary M, Maleta K, Manary M. A large-scale operational study of home-based therapy with ready-to-use therapeutic food in childhood malnutrition in Malawi. Matern Child Nutr. 2007;3(3).

48. WHO. WHO Child Growth Standards Length/height-for-age, weight-for-age, weight-for-length, weight-for-height and body mass index-for-age Methods and development Department of Nutrition for Health and Development. 2006.

49. Fabiansen C, Yaméogo CW, Iuel-Brockdorf AS, Cichon B, Rytter MJH, Kurpad A, et al. Effectiveness of food supplements in increasing fat-free tissue accretion in children with moderate acute malnutrition: A randomised 2 × 2 × 3 factorial trial in Burkina Faso. PLoS Med. 2017 Sep 1;14(9).

50. Millward DJ. A Protein-Stat Mechanism for Regulation of Growth and Maintenance of the Lean Body Mass. Nutr Res Rev. 1995 Jan;8(1):93–120.

51. Semba RD, Trehan I, Gonzalez-Freire M, Kraemer K, Moaddel R, Ordiz MI, et al. Perspective: The Potential Role of Essential Amino Acids and the Mechanistic Target of Rapamycin Complex 1 (mTORC1) Pathway in the Pathogenesis of Child Stunting. Advances in Nutrition. 2016 Sep 1;7(5):853–65.

52. Action Against Hunger. Guidelines: For the integrated management of severe acute malnutrition: In- and out-patient treatment | Action Against Hunger [Internet]. [cited 2020 Oct 22]. Available from: https://www.actionagainsthunger.org/publication/guildines-integrated-management-severe-acute-malnutrition-and-out-patient-treatment

53. Rolland-Cachera MF, Brambilla P, Manzoni P, Akrout M, Sironi S, del Maschio A, et al. Body composition assessed on the basis of arm circumference and triceps skinfold thickness: A new index validated in children by magnetic resonance imaging. American Journal of Clinical Nutrition [Internet]. 1997 [cited 2021 Jan 20];65(6):1709–13. Available from: https://pubmed.ncbi.nlm.nih.gov/9174464/

54. Lelijveld N, Seal A, Wells JC, Kirkby J, Opondo C, Chimwezi E, et al. Chronic disease outcomes after severe acute malnutrition in Malawian children (ChroSAM): a cohort study. Lancet Glob Health. 2016 Sep 1;4(9):e654–62.

55. Bourdon C, Bartels RH, Chimwezi E, Kool J, Chidzalo K, Perot L, et al. The clinical use of longitudinal bio-electrical impedance vector analysis in assessing stabilization of children with severe acute malnutrition. Clinical Nutrition. 2021 Apr 1;40(4):2078–90.

56. Bourdon C, Bartels RH, Chimwezi E, Kool J, Chidzalo K, Perot L, et al. The clinical use of longitudinal bio-electrical impedance vector analysis in assessing stabilization of children with severe acute malnutrition. Clinical Nutrition. 2020;0(0).

57. Iuel-Brockdorf AS, Dræbel TA, Fabiansen C, Cichon B, Christensen VB, Yameogo C, et al. Acceptability of new formulations of corn-soy blends and lipid-based nutrient supplements in Province du Passoré, Burkina Faso. Appetite [Internet]. 2015 [cited 2022 Feb 8];91:278–86. Available from: https://pubmed.ncbi.nlm.nih.gov/25913687/

58. Harris PA, Taylor R, Thielke R, Payne J, Gonzalez N, Conde JG. Research electronic data capture (REDCap)-A metadata-driven methodology and workflow process for providing translational research informatics support. J Biomed Inform. 2009;42(2):377–81.

59. StataCorp. Stata Statistical Software: Release 16. College Station, TX: StataCorp LLC; 2019.

60. Maleta K, Fan Y mei, Luoma J, Ashorn U, Bendabenda J, Dewey KG, et al. Infections and systemic inflammation are associated with lower plasma concentration of insulin-like growth factor I among Malawian children. 2020;(7):1–11.

61. Demidenko E, Stukel TA. Influence analysis for linear mixed-effects models. Stat Med [Internet]. 2005 Mar 30 [cited 2021 Oct 12];24(6):893–909. Available from: https://onlinelibrary.wiley.com/doi/full/10.1002/sim.1974

62. Yu H, Jiang S, Land KC. Multicollinearity in hierarchical linear models. Soc Sci Res. 2015 Sep 1;53:118–36.

63. Efron B, Tibshirani RJ. A n Introduction to the Bootstrap. An introduction to the bootstrap" Monographs on statistics and applied probability. 1993;1–436.
